# CAMKII as a therapeutic target for growth factor–induced retinal and choroidal neovascularization

**DOI:** 10.1172/jci.insight.122442

**Published:** 2019-03-21

**Authors:** Sadaf Ashraf, Samuel Bell, Caitriona O’Leary, Paul Canning, Ileana Micu, Jose A. Fernandez, Michael O’Hare, Peter Barabas, Hannah McCauley, Derek P. Brazil, Alan W. Stitt, J. Graham McGeown, Tim M. Curtis

**Affiliations:** 1 Wellcome-Wolfson Institute for Experimental Medicine and; 2 Advanced Imaging Core Technology Unit, Faculty of Medicine, Health and Life Sciences, Queen’s University of Belfast, Belfast, United Kingdom.

**Keywords:** Angiogenesis, Ophthalmology, Retinopathy, growth factors

## Abstract

While anti-VEGF drugs are commonly used to inhibit pathological retinal and choroidal neovascularization, not all patients respond in an optimal manner. Mechanisms underpinning resistance to anti‑VEGF therapy include the upregulation of other proangiogenic factors. Therefore, therapeutic strategies that simultaneously target multiple growth factor signaling pathways would have significant value. Here, we show that Ca^2+^/calmodulin-dependent kinase II (CAMKII) mediates the angiogenic actions of a range of growth factors in human retinal endothelial cells and that this kinase acts as a key nodal point for the activation of several signal transduction cascades that are known to play a critical role in growth factor–induced angiogenesis. We also demonstrate that endothelial CAMKIIγ and -δ isoforms differentially regulate the angiogenic effects of different growth factors and that genetic deletion of these isoforms suppresses pathological retinal and choroidal neovascularization in vivo. Our studies suggest that CAMKII could provide a novel and efficacious target to inhibit multiple angiogenic signaling pathways for the treatment of vasoproliferative diseases of the eye. CAMKIIγ represents a particularly promising target, as deletion of this isoform inhibited pathological neovascularization, while enhancing reparative angiogenesis in the ischemic retina.

## Abstract 2

CAMKII may provide an efficacious target to inhibit multiple angiogenic signaling pathways in the treatment of vasoproliferative diseases of the eye.

## Introduction

Aberrant angiogenesis is a pathological hallmark of several diseases, including rheumatoid arthritis and solid tumor growth (1, 2). In the retina, pathological angiogenesis underlies some of the major causes of blindness in developed countries, such as retinopathy of prematurity (ROP), proliferative diabetic retinopathy (PDR), and neovascular (wet) age‑related macular degeneration (nvAMD) (3). Increased levels of VEGF are thought to play a key role in the development of pathological angiogenesis in these conditions (4). Anti‑VEGF therapy has now become a mainstream treatment for nvAMD (5) and has been reported to have beneficial effects in other ocular angiogenic diseases (6, 7). However, despite the effectiveness of anti-VEGF therapy, many patients do not respond optimally to these drugs or they become refractory after prolonged treatment (8, 9). Resistance to anti-VEGF therapy is perhaps most easily explained by the involvement of other angiogenic mediators in driving the neovascular response, with previous work strongly implicating several other growth factors, including basic fibroblast growth factor (bFGF), insulin-like growth factor-1 (IGF-1), and hepatocyte growth factor (HGF) in regulating pathological angiogenesis in the eye (3, 10–14). These observations suggest that the identification of new therapeutic targets involved in orchestrating the complex proangiogenic effects of multiple growth factors could lead to efficacious new approaches for major blinding diseases.

Ca^2+^/calmodulin-dependent kinase II (CAMKII) is a serine/threonine protein kinase involved in translating intracellular Ca^2+^ signals into cellular responses (15). It consists of 4 different isoforms with distinct expression patterns, suggesting that isoform-specific interventions might be relatively tissue selective. In vascular endothelial cells, both the γ and δ isoforms have been found to be strongly expressed at both the mRNA transcript and protein level (16). Presently, we have only a limited understanding of the function of CAMKII in vascular endothelial cells, although studies to date have revealed a role for this kinase in modulating ion homeostasis, nitric oxide production, and agonist-induced changes in vascular permeability (17). We have previously reported that CAMKII acts as a key signaling molecule underlying VEGF-induced angiogenic activity in retinal endothelial cells in vitro (18), but whether it is involved in angiogenic signaling mediated by a broader range of growth factors remains unknown. Further validation of CAMKII as a potential therapeutic target for ocular angiogenic disorders also requires a clearer understanding of its contribution to retinal neovascularization in vivo, the downstream signaling pathways through which it mediates its proangiogenic effects, and the role of specific endothelial CAMKII isoforms. The aim of the current study was to address these issues using in vitro and in vivo models of retinal angiogenesis combined with pharmacological, siRNA knockdown, and whole-animal gene knockout approaches. Our data show that CAMKII is a potential target for the development of improved antiangiogenic therapies for the eye and that targeting either CAMKIIγ or -δ isoforms provides a potentially novel approach for differentially modulating angiogenic signaling through different growth factors in the retina.

## Results

### CAMKII mediates the angiogenic actions of a range of growth factors in retinal endothelial cells.

**Figure 1:**
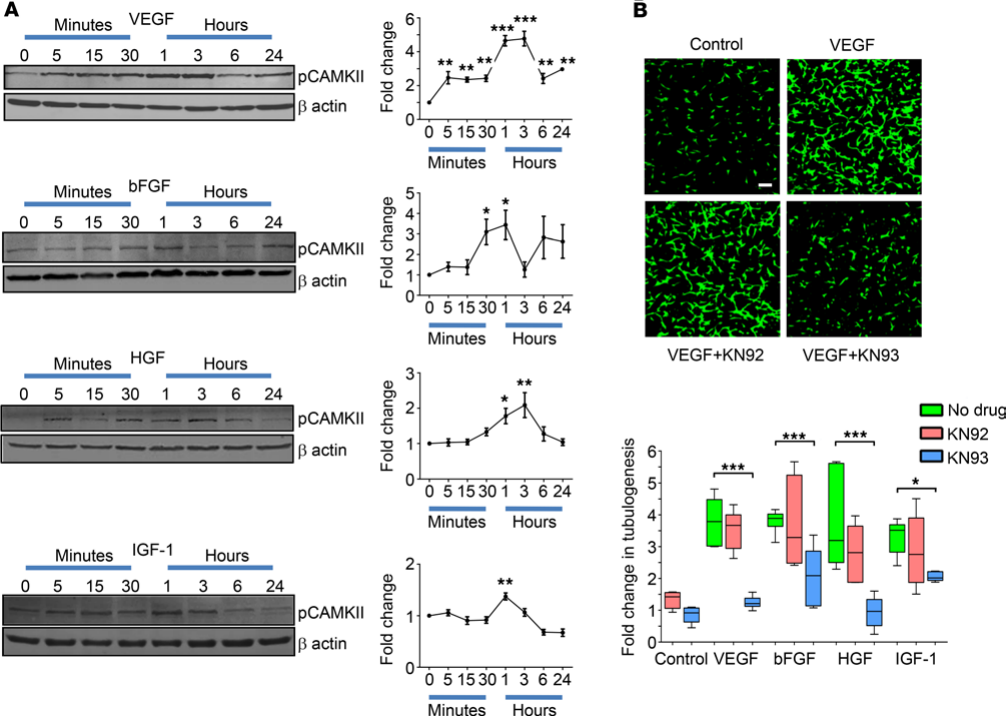
Ca^2+^/calmodulin-dependent kinase II (CAMKII) contributes to growth factor–induced retinal angiogenesis in vitro. (**A**) The time-dependent effects of various growth factors on the phosphorylation level of the CAMKII autoactivation site (T287) was investigated in human retinal microvascular endothelial cells (HRMECs) by Western blot analysis. Left*,* representative Western blots showing that VEGF, bFGF, HGF, and IGF-1 trigger CAMKII phosphorylation (pCAMKII) in HRMECs in a time-dependent manner. β-Actin was used as a loading control. Right, summary data calculated from the integrated density of the protein bands (normalized to β-actin) and expressed as a fold-change from untreated cells (time 0). Data represent mean ± SEM; *P < 0.05, **P < 0.01, ***P < 0.001 vs. time 0 based on ANOVA; *n* = 4 biological replicates. (**B**) Tubulogenesis assays were performed on HRMECs that were untreated or treated with various growth factors in the absence or presence of the CAMKII inhibitor KN93 (10 μM) or its inactive analogue KN92 (10 μM). Top, typical images showing the effects of KN93 and KN92 on VEGF-induced tube formation in HRMECs stained with calcein green. Scale bar: 100 μm. Bottom, box-and-whisker plots (min, max, 25th–75th percentile, median) showing that VEGF, bFGF, HGF, and IGF-1 stimulate HRMEC tubulogenesis in a CAMKII-dependent manner. *P < 0.05, ***P < 0.001 based on ANOVA; *n* = 4 biological and 3 technical replicates.

The wider contribution of CAMKII to growth factor–induced retinal angiogenic signaling in vitro was evaluated with focus on the ability of VEGF, bFGF, HGF, and IGF-1 to activate CAMKII in human retinal microvascular endothelial cells (HRMECs). Time‑dependent effects on the phosphorylation level of the CAMKII autoactivation site at T287 was assessed using Western blot analysis. Growth factor concentrations (25 ng/ml) were chosen to fall within the range reported in the vitreous and retina of patients with ocular neovascular disease (~5–50 ng/ml) (10, 19–21). VEGF, bFGF, HGF, and IGF-1 increased the amount of phosphorylated CAMKII, with peak levels differentially occurring between 1–3 hours after treatment (Figure 1A) and then dropping back toward basal levels within 24 hours. In contrast to these other growth factors, platelet-derived growth factor (PDGF) had no effect on CAMKII phosphorylation, thereby acting as a negative control for these experiments (Supplemental Figure 1; supplemental material available online with this article; https://doi.org/10.1172/jci.insight.122442DS1).

To directly study the role of CAMKII in eliciting angiogenic responses to VEGF, bFGF, HGF, and IGF-1, we used a tubulogenesis assay, in which endothelial cells form capillary-like tubes within a Matrigel matrix (22). All of the growth factors tested stimulated tubulogenesis by ~3- to 4-fold when compared with untreated control cells (Figure 1B; *P* < 0.001 for all growth factors vs. untreated controls). Treatment of HRMECs with the CAMKII inhibitor KN93 (10 μM), but not its inactive analogue KN92 (10μM), suppressed growth factor–induced tubulogenesis to levels that did not significantly differ from those of untreated control cells (Figure 1B; *P* > 0.05 for all growth factors plus KN93 vs. untreated controls). KN93 had no effects on tubulogenesis in control HRMECs in the absence of added growth factors (Figure 1B; *P* > 0.05).

**Figure 2:**
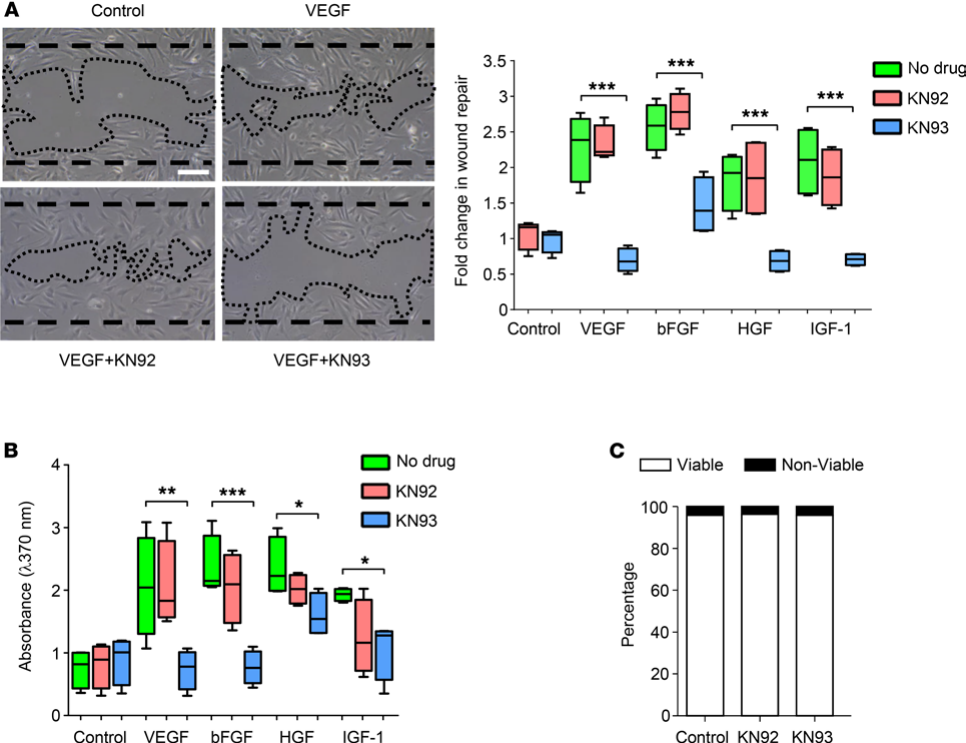
Pharmacological inhibition of Ca^2+^/calmodulin-dependent kinase II (CAMKII) blocks growth factor–induced migration and proliferation of human retinal microvascular endothelial cells (HRMECs) with no effect on cell viability. (**A**) Left, representative phase-contrast images of the migration scratch wound assay showing the extent of wound repair following stimulation of HRMECs with VEGF in the absence or presence of the CAMKII inhibitor KN93 (10 μM) or its inactive compound KN92 (10 μM). Dashed and dotted lines indicate wound edges at time 0 and 18 hours, respectively. Scale bar: 100 μm. Right, box-and-whisker plots (min, max, 25th–75th percentile, median) showing that VEGF-, bFGF-, HGF-, and IGF-1–induced wound repair was inhibited by KN93 but not KN92. (**B**) BrdU-ELISA cell proliferation assay. Box-and-whisker plots show the median values of BrdU absorbance for each treatment condition. Preincubation of HRMECs with 10 μM KN93 reduced the increase in DNA synthesis induced by VEGF, bFGF, HGF, and IGF-1. (**C**) Prolonged (24-hour) exposure of HRMECs to 10 μM KN93 had no effect on cell viability as measured using the Trypan blue exclusion assay. *P < 0.05, **P < 0.01, ***P < 0.001 based on ANOVA; *n* = 4 biological and 3 technical replicates for each assay.

The effects of CAMKII inhibition on growth factor–induced migration and proliferation in HRMECs were also assessed. VEGF, bFGF, HGF, and IGF-1 increased HRMEC migration and proliferation (Figure 2, A and B; *P* < 0.01 and *P* < 0.05 for all growth factors vs. untreated control cells for the 2 assays, respectively), and these effects were inhibited by KN93 but not KN92 (Figure 2, A and B; *P* > 0.05 for all growth factors plus KN93 vs. untreated controls in both assays). Consistent with our tubulogenesis findings, basal migration and proliferation were unaffected by KN93 in control HRMECs in the absence of added growth factors (Figure 2, A and B; *P* > 0.05). To ensure that KN93 did not affect cell viability, we performed Trypan blue exclusion assays, and prolonged exposure (24 hours) of HRMECs to this inhibitor had no effect on cell viability when compared with control and KN92‑treated cells (Figure 2C; *P* > 0.05). Taken together, the above experiments suggest that CAMKII activation represents a critical signaling step through which various growth factors initiate angiogenic activity in human retinal endothelial cells.

### VEGF induces the phosphorylation of several kinases in a CAMKII-dependent manner.

**Figure 3:**
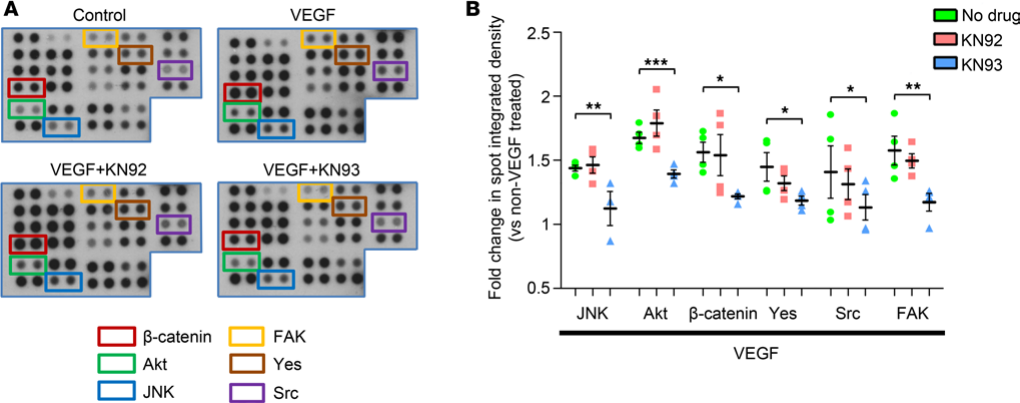
Phospho-proteomic analysis of human retinal microvascular endothelial cells (HRMECs) using the R&D Systems Human Phospho-Kinase array. HRMECs were treated with VEGF in the presence or absence of the Ca^2+^/calmodulin-dependent kinase II inhibitor KN93 (10 μM) or its inactive compound KN92 (10 μM) and the phosphorylation level of individual kinases represented on the array compared with untreated (control) cells. (**A**) Representative images showing a selected region of the phospho-kinase array for control cells and HRMECs exposed to VEGF with or without KN93 or KN92 treatment. Each kinase is spotted in duplicate and the location of JNK, Akt (S473), Yes, Src, FAK, and β-catenin is denoted using colored boxes. (**B**) Quantitative analysis of the spots was performed by densitometry and presented as fold change vs. control cells. Data represent mean ± SEM. *P < 0.05, **P < 0.01, ***P < 0.001 based on ANOVA; *n* = 2 biological replicates and 4 technical replicates per group.

We have previously reported that VEGF-induced phosphorylation of the proangiogenic protein, Akt (S473), is dependent on CAMKII activation in retinal endothelial cells (18). To gain a more thorough understanding of the downstream signaling pathways through which CAMKII regulates angiogenic activity, phospho‑kinase array experiments were conducted. HRMECs were exposed to VEGF (25 ng/ml) for 24 hours in the absence or presence of KN92 or KN93, and the phosphorylation levels of the individual kinases represented on the array were compared with untreated control cells. VEGF alone increased phosphorylation of 13 of 43 kinases included in the array (Supplemental Table 1). KN93, but not KN92, blocked VEGF-induced phosphorylation of JNK, Akt (S473), Yes, Src, and FAK, suggesting that these kinases are activated in a CAMKII‑dependent manner (Figure 3). In silico analyses using 3 different site-specific kinase-substrate prediction algorithms (Scansite 4.0, iGPS, and PhosphoSitePlus; refs. 23–25) indicated that phosphorylation of these proteins by CAMKII is likely to be indirect, suggesting the presence of intermediate kinases in the signal transduction pathway. Using the array, β‑catenin protein expression was increased in HRMECs exposed to VEGF, which was largely reversed by KN93 but not KN92 (Figure 3 and Supplemental Table 1). VEGF had no effect on HSP60 protein levels (Supplemental Table 1).

### Pharmacological inhibition of CAMKII blocks retinal neovascularization in vivo.

Thus far, our in vitro data supports the view that CAMKII may be a promising therapeutic target for the treatment of ocular neovascular disorders. To test this possibility further, we explored the effects of pharmacological inhibition of CAMKII on pathological retinal angiogenesis in vivo. The oxygen-induced retinopathy (OIR) mouse model was chosen for these studies because it is a well‑established model for studying pathological angiogenesis in the eye (26) and, similar to human ischemic retinopathies (e.g., ROP and PDR), neovascular pathology in this model involves multiple growth factors, including VEGF, bFGF, HGF, and IGF-1 (27–30).

**Figure 4:**
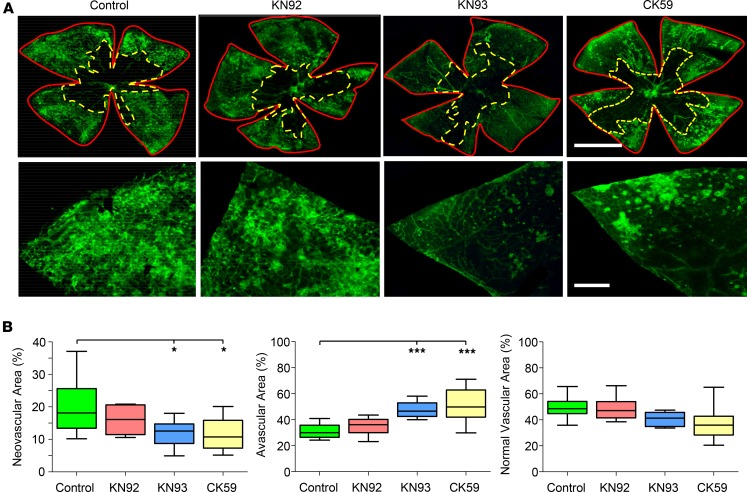
Pharmacological blockade of Ca^2+^/calmodulin-dependent kinase II (CAMKII) suppresses pathological angiogenesis in the ischemic retina. The murine oxygen-induced retinopathy (OIR) model was used to explore the role of CAMKII in retinal neovascularization in vivo. (**A**) Top panels**,** typical images of P17 flat-mounted retinas following OIR in a control (no drug) animal and mice intravitreally injected at P15 with KN92 (negative control for KN93), KN93, and CK59 (CAMKII inhibitors). Lectin staining (green) identifies the retinal vasculature. Red (solid) and yellow (dashed) lines demarcate total retinal areas and avascular areas, respectively, as defined using OIR Select software. Scale bar: 1.0 mm. Lower panels, higher-magnification images of selected areas of the flatmount preparations highlighting the differences in neovascular tuft formation among the treatment groups. Scale bar: 200 μm. (**B**) Box-and-whisker plots (min, max, 25th–75th percentile, median) of the neovascular, avascular, and normal vascular areas for the different groups expressed as a percentage of the total retinal area. *P < 0.05, ***P < 0.001 based on ANOVA; *n* = 10 mice per group.

Following OIR induction, intravitreal injection of mice at P15 with KN93 led to a significant reduction in neovascularization at P17 when compared with OIR control animals (Figure 4, A and B). We also tested the effects of a second structurally unrelated CAMKII inhibitor, CK59 (31). Like KN93, CK59 suppressed neovascularization in the OIR model (Figure 4, A and B). Central avascular and normal vascular areas were also quantified for the various treatment groups and compared with OIR control animals. Avascular areas were greater in OIR mice intravitreally injected with KN93 or CK59, but there was no change in normal vascular area with these drugs (Figure 4, A and B). Neovascular, avascular, and normal vascular areas in mice intravitreally injected with KN92 were similar to those measured in OIR control animals (Figure 4, A and B). These data suggest that CAMKII signaling makes a significant contribution to ischemia‑induced neovascularization in the retina in vivo.

*CAMKIIγand -δisoforms differentially regulate the angiogenic effects of different growth factors*. Our pharmacological data indicate that CAMKII is a key mediator of growth factor–induced retinal angiogenesis in vitro and in vivo, but they provide no information on the identity of the CAMKII isoforms involved. To address this issue, we employed an siRNA-based approach to evaluate the effects of knocking down expression of specific CAMKII isoforms on growth factor–induced retinal angiogenesis in vitro. We focused these studies on CAMKIIγ and -δ, since these are known to be the primary isoforms expressed in endothelial cells (16) and since previous work has shown that expression of CAMKIIα and -β in the retina is restricted to ganglion and amacrine cells (32, 33).

**Figure 5:**
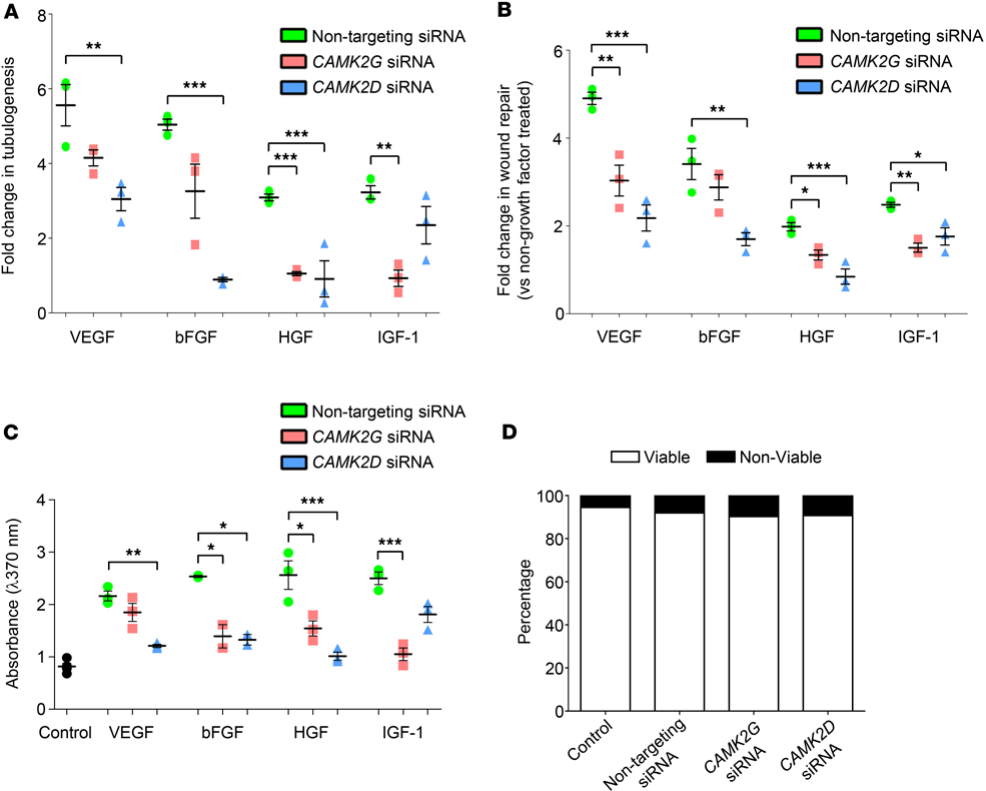
Ca^2+^/calmodulin-dependent kinase II (CAMKII) γ and -δ isoforms differentially regulate the angiogenic effects of different growth factors. (**A**) VEGF-, bFGF-, HGF-, and IGF-1–induced tubulogenesis in human retinal microvascular endothelial cells (HRMECs) transfected with nontargeting, *CAMK2G*, or *CAMK2D* siRNAs. (**B**) Quantitative evaluation of the effects of *CAMK2G* and *CAMK2D* silencing on growth factor–induced HRMEC migration determined using the scratch-wound assay. (**C**) BrdU-ELISA results showing the effects of *CAMK2G* and *CAMK2D* knockdown on VEGF-, bFGF-, HGF-, and IGF-1–induced cell proliferation. (**D**) Percentage cell viability data measured using the Trypan blue method for HRMECs transfected with nontargeting, *CAMK2G*, or *CAMK2D* siRNAs. Data represent mean ± SEM. *P < 0.05, **P < 0.01, ***P < 0.001 based on ANOVA; *n* = 3 biological and 3 technical replicates for each assay.

Knockdown of CAMKIIγ expression in HRMECs inhibited tube formation induced by HGF and IGF-1 when compared with cells transfected with nontargeting control siRNA (Figure 5A). In contrast, there were no significant changes in VEGF- and bFGF-induced tubulogenesis in cells treated with CAMKIIγ siRNA (Figure 5A). Knockdown of CAMKIIδ resulted in a different pattern of effects, suppressing tubulogenesis induced by VEGF, bFGF, and HGF, but not IGF-1 (Figure 5A). The extent to which individual growth factors stimulated tubulogenesis did not differ significantly between nontransfected HRMECs and cells transfected with nontargeting control siRNA (*P* > 0.05 in all cases).

To investigate further a differential role for CAMKIIγ and -δ isoforms in mediating the angiogenic activity of different growth factors, HRMEC migration and proliferation assays were performed. Results from these experiments were broadly consistent with the tubulogenesis assays, demonstrating a preferential involvement of CAMKIIγ in IGF-1– and CAMKIIδ in VEGF- and bFGF-induced angiogenic signaling, with both isoforms making a significant contribution to HGF-induced migration and proliferation (Figure 5, B and C). In these experiments, however, knockdown of CAMKIIδ did have a small but significant effect on IGF-1–induced migration (Figure 5B), and targeting CAMKIIγ reduced VEGF- and bFGF-induced migration and proliferation, respectively (Figure 5, B and C). In control comparisons, growth factor–induced migration and proliferation were similar in untreated and nontargeting control siRNA-treated cells (*P* > 0.05 for all growth factors tested), and no effects on cell viability were observed in any of the siRNA-transfected groups (Figure 5D). Overall, these findings suggest that the angiogenic effects of VEGF, bFGF, HGF, and IGF-1 rely on the activation of CAMKIIγ and -δ isoforms to differing extents in vitro.

**Figure 6:**
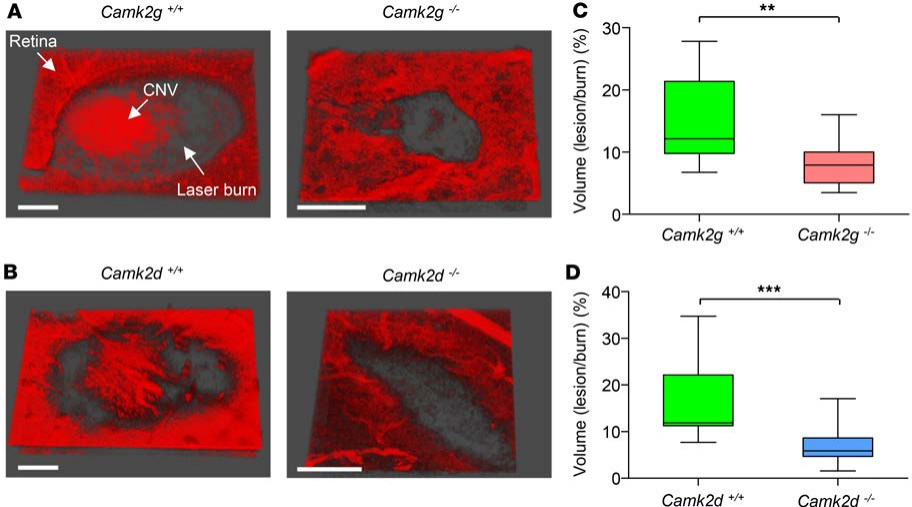
Laser-induced choroidal neovascularization (CNV) is reduced in CAMKIIγ- and -δ–KO mice. CNV lesions were generated using laser photocoagulation and quantified by multiphoton imaging of isolectin B4–stained retinal-choroidal flatmounts. (**A** and **B**) Representative 3-D images of isolectin B4–stained CNV lesions from CAMKIIγ and -δ WT and homozygous KO mice. Large CNV lesions are clearly evident within laser burns of the WT mice. Scale bars: 10 µm. (**C** and **D**) Box-and-whisker plots (min, max, 25th–75th percentile, median) comparing CNV lesion volumes (expressed as a percentage of the total burn volume) between CAMKIIγ and -δ WT and KO mice. KO animals exhibited significantly less CNV than their WT counterparts. **P < 0.01 and ***P < 0.001 based on 2-tailed Student *t* test; *n* = 5–10 animals per group.

*Genetic deletion of CAMKIIγand -δisoforms reduces laser-induced choroidal neovascularization (CNV) in vivo*. Building on our in vitro data, the role of CAMKIIγ and -δ isoforms was evaluated in vivo using the laser-induced mouse model of CNV (34). In the neovascular form of AMD, Bruch’s membrane damage is associated with the formation of CNV lesions (3). Postmortem evaluation of retinal flatmounts stained with isolectin B4 demonstrated a significant reduction in CNV lesion volumes in CAMKIIγ- and -δ–KO mice when compared with their WT counterparts (Figure 6).

**Figure 7:**
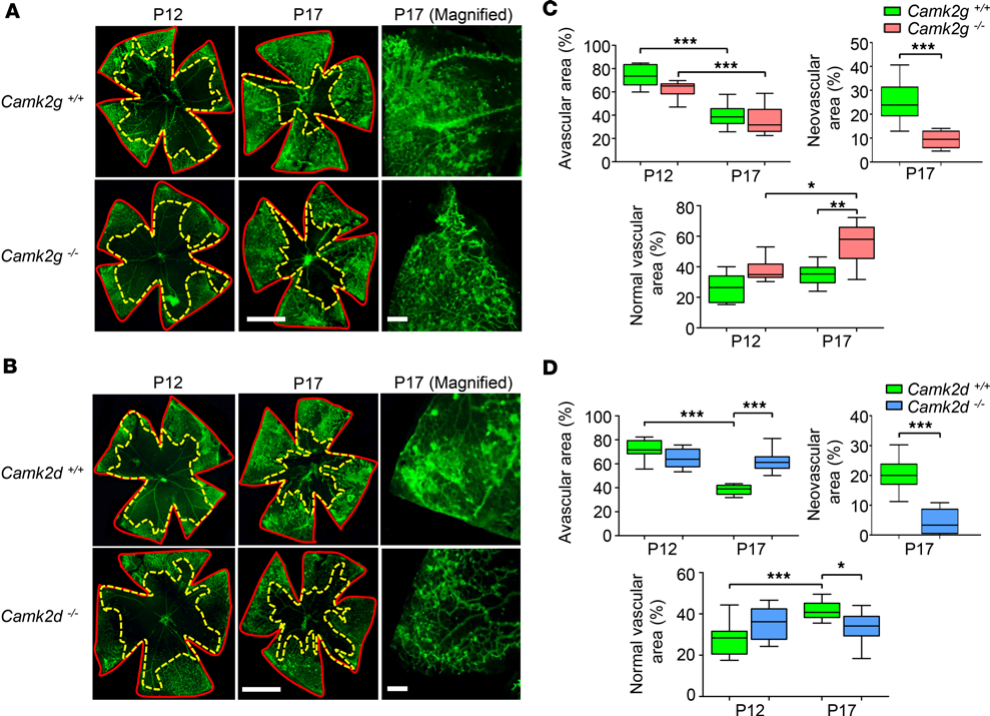
Oxygen-induced retinopathy (OIR) in CAMKIIγ- and -δ–KO mice. Pups were exposed to hyperoxia (75% O_2_) from P7–P12, followed by 5 days in room air to induce vascular insufficiency and preretinal neovascularization. Some mice were euthanized at P12 to assess the degree of retinal vascular regression. (**A** and **B**) Left and middle, isolectin B4–stained flat‑mounted retinas from CAMKIIγ and -δ WT and homozygous KO OIR mice at P12 and P17. Red (solid) and yellow (dashed) lines demarcate total retinal areas and avascular areas, respectively. Scale bars: 1.0 mm. Right, magnified regions of the P17 retinal flatmounts. Scale bars: 200 μm. (**C** and **D**) Box-and-whisker plots (min, max, 25th–75th percentile, median) of avascular, neovascular, and normal vascular areas expressed as a percentage of the total retinal area for the various cohorts of mice at P12 and P17. Neovascularization was absent at P12 and, therefore, has not been plotted. *P < 0.05, **P < 0.01, ***P < 0.001 based on ANOVA (avascular and normal vascular) and 2-tailed Student *t* test (neovascular). Data is derived from a minimum of 10 mice per group.

*CAMKIIγ-and -δ–KO mice respond differently to ischemic retinopathy in vivo*. The OIR model was used to further substantiate a role for CAMKIIγ and -δ isoforms in pathological retinal angiogenesis in vivo. CAMKIIγ- and -δ–KO mice and their WT controls displayed a similar sensitivity to hyperoxic vasoregression, with no significant differences observed in avascular or normal vascular areas at P12 (Figure 7). After 5 days of ischemic hypoxia (P17), the degree of OIR in CAMKIIγ and -δ WT mice (Figure 7) was comparable with control animals in our previous pharmacological studies (avascular, neovascular, and normal vascular areas of 40%, ~20% and ~40%, respectively). When compared with their respective WT controls, CAMKIIγ- and -δ–KO mice exhibited a substantial decrease in retinal neovascularization (Figure 7). A marked difference, however, was noted with regard to changes in the avascular and normal vascular areas between the 2 groups of KO animals. In CAMKIIγ KOs, avascular areas were similar, but normal vascular areas were significantly increased when compared with their WT littermates (Figure 7, A and C). In CAMKIIδ KOs, avascular areas were increased and normal vascular areas reduced in comparison with their WT controls (Figure 7, B and D). In separate studies, adult CAMKIIγ- and -δ–KO mice displayed no overt changes in the architecture of the retinal vascular network (Supplemental Figure 2) or in the gross morphology of the retina (Supplemental Figure 3) when compared with their WT littermate controls. These OIR data show that deletion of CAMKIIγ and -δ isoforms elicit different outcomes during ischemic retinopathy in vivo. Genetic ablation of CAMKIIγ inhibits preretinal neovascularization, while concomitantly enhancing vascular recovery within the ischemic tissue. Deletion of CAMKIIδ suppresses preretinal neovascularization, but also has a detrimental impact on reparative angiogenesis in the retina during the hypoxic phase of the OIR model.

To investigate whether the differential effects of knocking out CAMKIIγ and -δ during OIR might relate to differences in the normal cellular localization and expression of these isoforms in the retina, we performed dual RNAscope in situ hybridization assays on retinal sections from P15 C57BL/6 control (normoxic) and OIR mice. Sections were colabeled with RNAscope probes for either CAMKIIγ (*Camk2g*) or CAMKIIδ (*Camk2d*) together with claudin-5 (*Cldn5*) to positively identify the vascular endothelium. In control retinas, *Camk2g* and *Camk2d* exhibited similar distribution patterns, being localized not only to the vascular endothelium, but also to cells within the ganglion cell layer (GCL), inner nuclear layer (INL), and outer nuclear layer (ONL) (Supplemental Figure 4). *Camk2d*, but not *Camk2g*, mRNA expression was found to be significantly upregulated during OIR (Supplemental Figure 4). However, no obvious changes in the cellular localization of these CAMKII isoforms were observed (Supplemental Figure 4).

## Discussion

Anti-VEGF drugs have revolutionized the management of patients with nvAMD, and they have shown great promise for the treatment of other ocular angiogenic diseases (35). Unfortunately, blockade of VEGF is not universally effective, and a significant subset of patients fail to respond or become refractory to treatment. Pathological angiogenesis in the eye involves the concerted actions of multiple growth factors, and more effective antiangiogenic therapies may need to go beyond targeting VEGF alone (36–38). Herein, we have identified CAMKII as a common signaling step that is evoked by multiple proangiogenic growth factors, irrespective of whether the pathological response was preretinal or subretinal. Our data suggest that this kinase could represent an important target for the development of improved antiangiogenic therapies for the eye.

The current study has demonstrated that CAMKII is activated in HRMECs by various growth factors implicated in vasoproliferative diseases of the eye and that its pharmacological inhibition prevents angiogenic responses induced by bFGF, HGF, and IGF-1, as well as VEGF. Direct activation of CAMKII occurs upon an initial increase in intracellular Ca^2+^ and Ca^2+^/calmodulin binding (15), and most growth factors that signal through receptor tyrosine kinases are known to raise intracellular Ca^2+^ via the phospholipase Cγ/IP_3_/Ca^2+^ signaling pathway (39). Indeed, a number of studies have shown that VEGF, bFGF, HGF, and IGF-1 can trigger Ca^2+^ mobilization from IP_3_-sensitive Ca^2+^ stores and Ca^2+^ entry across the plasma membrane in retinal and other types of vascular endothelial cells (18, 40, 41). We have previously reported that VEGF-induced retinal angiogenesis in vitro is prevented by treatment with the CAMKII inhibitor, KN93 (18), and the present study extends this work by showing that other important growth factors converge on CAMKII to mediate their angiogenic activity. Previous studies have demonstrated a requirement for CAMKII in growth factor–induced vascular smooth muscle cell (SMC) migration and proliferation (42, 43), while it may also regulate cancer cell proliferation, motility, and metastasis (44). The exact mechanisms through which CAMKII modulates cell migration and proliferation have yet to be fully explored, although recent work has suggested that CAMKII-dependent activation of the Akt, MAPK, and CREB pathways may be involved (45–47).

In an effort to better understand the downstream signaling molecules through which CAMKII regulates angiogenic activity in HRMECs, we used a phospho-kinase antibody array to identify kinases that are differentially phosphorylated by VEGF in the absence or presence of the CAMKII inhibitor KN93. Several kinases were phosphorylated by VEGF in a CAMKII-dependent manner, including JNK, Akt, Yes, Src, and FAK. These have all previously been shown to contribute to retinal angiogenic responses in vitro and pathological neovascularization in the retina in vivo (48–52). Based on our in silico analyses, these proteins do not appear to be direct substrates of CAMKII’s catalytic activity, suggesting that intermediate kinases are most likely responsible for their phosphorylation in response to CAMKII activation. Phosphorylation of JNK, Akt, and FAK downstream to CAMKII activation has been reported previously (18, 53, 54), but we are unaware of any earlier studies showing that CAMKII activity is required for growth factor–induced phosphorylation of the Src family kinases Yes and Src. KN92, the kinase-inactive control for KN93, had no effect on VEGF‑induced phosphorylation of JNK, Akt, Yes, Src, and FAK. In addition, a recent screen of KN93 against a broad range of human protein kinases has shown that this drug does not directly affect the activity of any of these kinases (55). Thus, it seems unlikely that the results from our phospho-kinase arrays can be explained on the basis of any nonspecific actions of the KN93 compound. Our array studies also revealed a CAMKII‑dependent increase in β‑catenin protein levels in HRMECs treated with VEGF. CAMKII‑dependent upregulation of β-catenin protein expression has been reported in other cell types (56), and this protein has been shown to promote endothelial cell angiogenic activity by regulating the expression of the cell cycle protein cyclin E2 (57). Overall, our phospho-kinase array studies suggest that CAMKII stimulates angiogenic activity by acting as a key nodal point for the activation of several signal transduction cascades that play a critical role in regulating growth factor–induced angiogenesis.

Up to now, an important question that has remained unanswered is whether CAMKII contributes to pathological angiogenesis in vivo. We have demonstrated that 2 structurally distinct CAMKII inhibitors, KN93 and CK59, suppress ischemia‑induced neovascularization in the OIR model. Furthermore, these drugs were found to block neovascularization without exerting any obvious effects on the preexisting vessels of the retina. It should be noted, however, that KN93 and CK59 are not isoform selective (31, 58), and from a therapeutic perspective, widespread blockade of all CAMKII isoforms in the retina would be expected to have a detrimental impact on retinal function. A splice variant of CAMKIIα, for example, has been implicated in mediating neuroprotection in retinal ganglion cells following glutamate excitotoxicity (59). Targeting of CAMKII for the treatment of retinal neovascular disease is, therefore, likely to require an isoform-specific approach. As an important step toward this goal, we determined the contribution of individual endothelial CAMKII isoforms (γ and δ) to retinal angiogenesis in vitro and in vivo using molecular genetic strategies.

Our siRNA-based studies revealed that both CAMKIIγ and -δ are involved in growth factor–induced retinal angiogenesis in vitro, although they appear to be linked to distinct signaling pathways. CAMKIIγ was required for IGF-1– and CAMKIIδ was required for VEGF- and bFGF-induced angiogenesis, with both isoforms contributing to the angiogenic effects of HGF. To the best of our knowledge, this is the first study to show that different growth factors can exert their actions by signaling through distinct CAMKII isoforms. Our data provide a basis for more detailed studies investigating the mechanisms through which different growth factors activate specific CAMKII isoforms in vascular endothelium.

The reductions in growth of laser-induced CNV lesions in CAMKIIγ- and -δ–KO mice represent key findings of this study since this murine model is widely used to reproduce key aspects of nvAMD (60). Genetic ablation of both isoforms equally reduced CNV, whereas genetic ablation of CAMKIIγ and -δ isoforms elicited differing effects in OIR (widely used model of ROP), although both contributed to pathological angiogenesis. Specifically, in the OIR model, physiological revascularization of the ischemic tissue was enhanced in CAMKIIγ- but inhibited in CAMKIIδ-KO mice. Our results in mice lacking CAMKIIγ, which siRNA studies showed was coupled with IGF‑1 signaling in HRMECs, bear strong resemblance to those previously reported in OIR mice with endothelial cell–specific deletion of the IGF-1 receptor (61). Similarly, KO of CAMKIIδ, which was associated with bFGF signaling in HRMECs, produced effects in the OIR model comparable with those previously described in mice with targeted deletion of endothelial cell FGF receptor signaling (28). To date, there have been no studies examining the consequences of endothelial cell–specific deletion of VEGF and HGF signaling in the OIR model. Since we used global KOs of CAMKIIγ and -δ isoforms, we cannot completely rule out the possibility that the differential effects of knocking out these isoforms on revascularization of the OIR retina may have resulted from changes at the level of the retinal neurons and glial cells. Retinal neurons, for instance, have been shown to play an important role in modulating regenerative angiogenesis in the OIR model (62). In the present study, we have shown that, in addition to being localized to the vascular endothelium, CAMKIIγ and -δ are also expressed in the nonvascular cells of the retina with distribution patterns that are similar in both normally perfused and ischemic retina. These observations would suggest that the reasons why genetic ablation of CAMKIIγ and -δ exert different effects in the OIR model cannot be simply explained on the basis of differences in the localization patterns of these isoforms. Interestingly, we did find that the expression levels of the 2 isoforms respond differently to ischemia (*Camk2d* mRNA was upregulated, while *CamK2g* remained unchanged). Nevertheless, these results are difficult to reconcile with the opposing effects of these isoforms on reparative angiogenesis in the ischemic retina. Future work directed toward identifying the main substrates of CAMKIIγ and -δ in the nonvascular cells of the retina may provide a useful starting point in attempting to unravel why deletion of these 2 isoforms exerts different OIR responses.

In summary, this study demonstrates the critical role of CAMKII in retinal angiogenesis. Our data support the idea that targeting of either CAMKIIγ or -δ isoforms in the retina could provide a means of inhibiting the effects of multiple angiogenic factors, offering potential advantages over current anti-VEGF approaches. We have shown that both isoforms are potential therapeutic targets for the treatment of CNV. For ischemic retinopathies, CAMKIIγ appears to be the most promising therapeutic target, as deletion of this isoform inhibited neovascular growth while enhancing revascularization of the ischemic retina. In contrast, genetic ablation of CAMKIIδ not only suppressed pathological retinal neovascularization, but also had a detrimental effect on reparative angiogenesis. Since CAMKIIγ appears to mediate angiogenesis independently of VEGF, targeting of this isoform could potentially improve clinical outcomes when used in combination with current anti-VEGF therapies and provide an alternative treatment for patients who fail to respond or become refractory to these drugs. CAMKIIγ and -δ have been identified as important targets for the treatment of a number of cardiovascular diseases (17, 63, 64), and our work strengthens the rationale for the development of selective inhibitors against these isoforms for selected retinopathies.

## Methods

### Drugs and solutions.

Recombinant VEGF_165_, bFGF, HGF, IGF-1, and PDGF were obtained from R&D Systems. The CAMKII inhibitor KN93 and its inactive analogue KN92 were purchased from Santa Cruz Biotechnology Inc. CK59 was obtained from Calbiochem. Stock solutions of growth factors were prepared in PBS, while pharmacological agents were dissolved in DMSO. The DMSO concentration in the assays never exceeded 0.1% (vol/vol), a concentration that was found not to influence the results of any of the assays.

### Cell culture.

Primary HRMECs were purchased from Cell Systems and grown on 1% gelatin-coated culture dishes in Cell Systems Corporation (CSC) complete medium (Cell Systems; 4ZO-500) containing 50 mg/ml primocin (InvivoGen). Cells were incubated at 37°C in a humidified atmosphere containing 5% CO_2_ and used at passages 6–8. Unless otherwise stated, experiments were repeated a minimum of 4 times using different batches of HRMECs, with 3 technical replicates per batch of cells.

### Immunofluoresence.

The endothelial characteristics of HRMECs were confirmed by immunolabeling studies. Cells were seeded onto zero thickness glass coverslips (Scientific Laboratory Supplies) and fixed in 4% paraformaldehyde (PFA) at room temperature for 20 minutes prior to incubating in blocking and permeabilization buffer (PB; 1% donkey serum and 0.05% Triton-X 100 in PBS) for 1 hour at room temperature. HRMECs were then washed in PBS for 1 hour prior to incubation with the following primary antibodies overnight at 4ºC: β-catenin (1:50; Santa Cruz Biotechnology Inc., catalog No Sc-7963), CD31 (1:50; Santa Cruz Biotechnology Inc., catalog No Sc-8306), vimentin (1:50; Dako, Agilent technologies, catalog M0725), von Willebrand factor (vWF; 1:50; Abcam, catalog Ab75117), CD105 (1:50; Dako catalog M3527), CD14 (1:50; Santa Cruz Biotechnology Inc., catalog Sc-6999) and α-smooth muscle actin (1:50; α-SMA; Dako, cataog M0851). Cells were then washed and incubated with appropriate secondary antibodies (1:500 donkey anti-mouse [A21202] or anti-rabbit Alexa Fluor488 [A21206]; Invitrogen) for 1 hour at room temperature. After washing, HRMECs were mounted onto Surgipath microscope slides (Leica Biosystems) with Vectashield containing the nuclear stain DAPI (Vector Laboratories Inc.). Images were captured using a Nikon Eclipse Ti-U inverted C1 confocal microscope. HRMECs were positive for the endothelial cell markers vWF, CD31 (PECAM-1), CD105 (endoglin), β-catenin, and vimentin (Supplemental Figure 5). In contrast, they were negative for markers of α-SMA and macrophages (CD14) (Supplemental Figure 5).

### Western blot analysis.

Cells were lysed in RIPA buffer supplemented with a protease inhibitor cocktail (Halt Protease Inhibitor Cocktail, EDTA-Free; 100×; 87785; Thermo Fisher Scientific). Supernatants were cleared by centrifugation at 16,000 *g* for 10 minutes (4°C), and protein concentration were determined using a BCA protein assay kit (Thermo Fisher Scientific). Protein samples (30 μg) were separated on 10% SDS-PAGE gels, transferred to PVDF membranes, and probed with polyclonal rabbit antibodies raised against phospho‑CAMKII (T287; 1:500; Abcam, catalog Ab138392) and mouse monoclonal anti–β-actin antibodies (1:10,000; Cell Signaling Technology, catalog 8H10D10, #3700). After washing, membranes were incubated with appropriate goat secondary antibodies (1:20,000) against rabbit or mouse IgG (IRDye 680 and IRDye 800; Li-COR). Immunoreactive bands were detected using a Li-COR Odyssey system. All western blot data shown are representative of at least 3 separate individual experiments.

### Tubulogenesis assay.

In this assay, endothelial cells form capillary-like tubes within a Matrigel matrix (22). HRMECs (1 × 10^5^ cells) were resuspended in CSC complete media containing 50% growth factor–reduced Matrigel (BD Biosciences) and 50 μl aliquots spotted onto 24-well plates. After polymerization, spots were covered in CSC complete media containing test substances and incubated at 37°C for 24 hours. Cells were then labeled with Cell Trace Calcein Green AM (0.5 μg/ml for 30 minutes; Invitrogen), and endothelial tube–like structures were imaged using fluorescence microscopy (Nikon Eclipse TS100). Tubulogenesis was quantified by measuring the tube area in 5 randomly selected low‑power fields (magnification ×10) from each well using NIS Elements software (Nikon), and data were presented relative to untreated control cells.

### Scratch wound migration assay.

HRMECs were grown to 80% confluence in CSC complete medium on 1% gelatin-coated 6‑well plates. A single uniform scratch wound that crossed each well was then performed using a sterilized 200 μl pipette tip. Following injury, cells were washed, and media containing test compounds were added. Scratch wound regions were imaged 0 and 18 hours after wounding using a phase contrast microscope equipped with a digital camera (Nikon Eclipse TS100; E5400 camera; Nikon). Wound repair was calculated by subtracting the total wound area at 18 hours from the total wound area at 0 hours using ImageJ (NIH) software (65), and the data were normalized to untreated control cells. All scratch wound assays were performed in the presence of 5-fluorouracil (1 mM) to prevent cell proliferation.

### Proliferation assay.

HRMECs were seeded at a density of 1 × 10^4^ cells/well in 96-well plates and cultured for 24 hours in CSC complete media. Media was then replaced with fresh CSC media containing test substances, and the cells were incubated for a further 24 hours. BrdU was then added and its incorporation into HRMEC DNA measured using a colorimetric cell proliferation ELISA assay (Roche Diagnostics).

### Cell viability.

HRMECs were grown to 80% confluency in CSC complete media on 1% gelatin-coated 24‑well plates and treated with test substances for 24 hours. Cells were then collected by trypsinization, washed in PBS, and stained with 0.2% Trypan blue. The proportion of viable cells was determined from hemocytometer counts.

### Phospho-kinase arrays.

Phospho-antibody array analysis was carried out using the Proteome Profiler Human Phospho-Kinase Array (R&D Systems) according to the manufacturer’s instructions. HRMECs were cultured in 1% gelatin–coated T75 flasks in CSC complete media and exposed to test substances for 24 hours. Cells were lysed with Lysis Buffer 6 (R&D Systems) and agitated for 1 hour at 4°C. Cell lysates were clarified by microcentifugation at 14,000 *g* for 5 minutes, and protein concentration were determined using a BCA protein assay kit (Thermo Fisher Scientific). Array membranes were blocked with Array Buffer 1 (R&D Systems) and incubated overnight at 4°C with 600 μg of cell lysate. The membranes were washed to remove unbound proteins and then incubated with biotinylated detection antibodies and streptavidin-HRP. Chemiluminescent detection reagents were applied to detect spot densities. Array images were analyzed using ImageJ software (65). Array spots were background subtracted and normalized to positive control spots on each membrane to enable comparisons across the different treatment groups. The integrated density of duplicated spots representing each phospho-kinase protein was determined, and data were presented as the fold change compared with the untreated control group. The phospho-antibody array experiment was repeated twice, comprising 2 biological and 4 technical replicates per treatment group.

### siRNA transfections.

HRMECs were transfected with Dharmacon ON-TARGETplus SMARTpool siRNAs specifically targeting human *CAMK2G* (catalog L-004536-00) or *CAMK2D* (catalog L-004042-00) isoforms for 24 hours using DharmaFECT reagent (Thermo Fisher Scientific). Dharmacon ON-TARGETplus siCONTROL Nontargeting siRNA (catalog D-001810-10) was used as control siRNA. Quantitative PCR (qPCR) was performed to determine knockdown efficiency as previously described (66). Primers (Integrated DNA Technologies) were designed to amplify human *CAMK2G* (forward primer 5′-TCCTGTATATCCTCCTGGT-3′, reverse primer 5′-CATCTGGTTGATCAAGTTC-3′) and human *CAMK2D* (forward primer 5′-GGATCTGTCAACGTTCTACT-3′, reverse primer 5′-TGTGGATTACAGTAGTTTGG-3′), which were quantified relative to *GAPDH* (human, forward primer 5′-GAGTCAACGGATTTGGTCGT-3′, reverse primer 5′-GACAAGCTTCCCGTTCTCAG-3′). Cells were transfected with 25 nM siRNA, a concentration that was found to consistently produce ≥80% transcript knockdown for each CAMKII isoform and where knockdown of *CAMK2G* had no effect on *CAMK2D* mRNA expression and vice versa (Supplemental Figure 6).

### Animals.

For pharmacological experiments, C57BL/6 mice were purchased from Harlan Laboratories. CAMKIIγ-KO (67) and CAMKIIδ-KO (68) mice were provided by Prof Eric Olson (University of Texas Southwestern Medical Center, Dallas, Texas, USA) and Johannes Backs (University of Heidelberg, Heidelberg, Germany). Heterozygous mice (^+/–^) were bred to produce CAMKIIγ and CAMKIIδ homozygous KO (^–/–^) and WT (^+/+^) mice. Mice were housed in standard microisolator cages in the Biological Services Unit at Queen’s University of Belfast and provided food and water ad libitum. Numbers of mice used for each experiment are indicated in the figure legends.

*Genotyping of CAMKIIγ-and CAMKIIδ-KO mice*. Genomic DNA was extracted from ear biopsies and amplified with the REDExtract-N-Amp Tissue PCR Kit (MilliporeSigma) using *Camk2g* and *Camk2d* primer mixes (Eurofin Genomics) before being loaded on to 1.5% agarose gel. The *Camk2g* primer mix (5′-CAC TAG TGC ACA AAG CCT TTT CAA-3′, 5′-ACT TGG GGA GTT GGT TCT CTT TTC-3′ and 5′-TAC ACG GTA AAT GCC TCA CAT ACG-3′) amplified a 207 bp WT band and 413 bp homozygous KO band. The *Camk2d* primer mix (5′-GAG AAG GGC GAA GAC GTG ACA G-3′, 5′-GAT GAG AGA CCA CTC GAA GCT C-3′ and 5′-GCC AAA GGA CAT ATC ACA CTG G-3′) amplified a 282 bp WT band and 600 bp homozygous KO band. A representative genotyping gel image of DNA from ear biopsies of CAMKIIγ and CAMKIIδ WT and homozygous KO mice is shown in Supplemental Figure 7A.

### Reverse transcription PCR (RT-PCR) amplification.

Total RNA was extracted from flash-frozen pairs of retinas from CAMKIIγ and CAMKIIδ WT and homozygous KO mice using a Qiagen RNeasy Mini Kit. Total RNA was quantified using a NanoDrop One spectrophotometer (Thermo Fisher Scientific), and cDNA was synthesized from 1 ng of total RNA using a High Capacity cDNA Reverse Transcription kit (Thermo Fisher Scientific) primed with a mixture of random primers. The cDNA RT products were amplified with *Camk2g*-, *Camk2d*-, and *Actn1-specific* (Actinin-1–specific) primers using a REDExtract-N-Amp PCR ReadyMix kit (MilliporeSigma). Primer sequences were as follows: *Camk2g*, forward primer, 5′-ACGACTACCAGCTTTTCGAGG-3′, and reverse primer, 5′-TTTGCAGCATATTCCTGC GT-3′; *Camk2d*, forward primer, 5′-CGGTTCACCGACGAGTATCA-3′, and reverse primer, 5′-TGGCAGCATACTCTTGTCCAG-3′; and *Actn1*, forward primer, 5′-TGCCTCAT CAGCTTGGGTTA-3′, and reverse primer, 5′-AAGTCGATGAAGGCCTG GAA-3′. PCR amplified products were resolved on 2.5% agarose gels and visualized using Midori Green nucleic acid stain. RT-PCR experiments confirmed retinal KO of *Camk2g* and *Camk2d* in the respective KO mouse strains (Supplemental Figure 7B).

### OIR model.

Ischemic retinopathy was induced in C57BL/6, WT, and gene-KO mice according to the protocol of Smith et al. (69). Briefly, P7 mice and their nursing mothers were exposed to 75% oxygen (OxyCycler; Biospherix Ltd.) for 5 days to induce vasodegeneration and atrophy of the central retinal capillary beds. At P12, mice were removed from the oxygen chamber and returned to normal room air to induce vascular insufficiency and ischemia‑driven neovascularization, a response that reaches a maximum around P17 (70). To visualize the degree of retinal vascular regression in the different groups of animals, some mice were euthanized at P12 and their eyes enucleated. This was important for studies using gene-KO mice to ensure that any changes observed between groups at P17 could not be attributed to differences in the initial levels of retinal ischemia produced at P12. For CAMKII inhibitor studies, mice were intravitreally injected with test substances (1 μl) at P15 using a 34-G bevelled needle (Nanofil; World Precision Instruments) under general anaesthesia (60 mg/kg ketamine + 6 mg/kg xylazine, i.p.). P15 was chosen for the intravitreal injections since preretinal neovascularization in the OIR model begins around this time point (71). Reported drug concentrations represent the estimated end vitreal concentrations, calculated assuming a vitreal volume of ~5 μl for a P15 mouse eye (72). One eye per mouse was treated with the contralateral untreated eye serving as an internal control. At P17, animals treated with CAMKII inhibitors, controls, WT, and gene-KO mice were sacrificed, and their eyes were collected for quantification of retinal neovascular, ischemic, and normally vascularized areas.

### Preparation and quantitative assessment of retinal flatmounts from OIR animals.

Enucleated eyes were immersion fixed in 4% PFA for 2 hours and washed extensively. Retinas were isolated and the vasculature fluorescently stained with biotin‑labeled isolectin B4 (1:50; MilliporeSigma) and Alexa Fluor Streptavidin 488 (1:500; Invitrogen). Retinas were then flat-mounted and imaged using a fluorescence microscope (Nikon Eclipse TS100). Neovascular, avascular and normal vascular areas were quantified as a percentage of the total retinal area using OIR Select software developed within our laboratory. This ImageJ-based plugin semiautomates the analysis of OIR images using 4 user-defined thresholding values based on Huang (73) (whole retina) and Otsu (74) (neovascular, avascular, and normal vascular areas) thresholding methods. Quantification of normal vascular area has not been widely reported in OIR studies, to date. The OIR Select software calculates the normal vascular area by subtracting the neovascular area from the total vascular area and expressing this as a percentage of the total retinal area. This end-point provides an indication of the degree of physiological revascularization across the entire retina during OIR. The OIR Select software and detailed user guide is available in the Supplemental Data 2.

### RNAscope in situ hydridization and image analysis.

Manual RNAscope 2.5 HD Duplex assays were performed on formalin-fixed paraffin‑embedded sections (5-μm thickness) from P15 control (normoxic) and OIR mouse retinas according to the manufacturer’s protocols (ACDBio; Doc #322452-USM and #322500-USM). The following sets of RNAscope probes were used in this study: (i) Mm-Camk2g-C2 (Cat #522071-C2) together with Mm-Cldn5 (Cat #491611) and (ii) Mm-Camk2d-O1 (Cat #508941) together with Mm-Cldn5-C2 (Cat #491611-C2). Slides were lightly counterstained with Gill’s hematoxylin solution and mounted in VectaMountTM (Vector Laboratories Inc.). Images were acquired using a Nikon E400 microscope with ×40 or ×60 air objectives. *Camk2g* and *Camk2d* dot quantification (×60 magnification) in claudin-5 (*Cldn5*)-positive vascular endothelial cells, the retinal GCL, INL, and ONL was performed using ImageJ (65). Five images were obtained per slide from each mouse, where each slide contained 2–3 retinal sections. For each image, dots were quantified in 3 randomly selected regions of interest per retinal region. Data are summarized as the average number of dots per mm^2^.

### CNV model.

CNV lesions were created as previously described (75). In brief, WT and gene-KO mice (6 months old) were anesthetized, and rupture of Bruch’s membrane-choroid was achieved by laser photocoagulation (Haag Streit BM 900 Slit Lamp and Argon laser) using burns of 50 μm spot size (0.05-second duration, 250 mW) approximately 2 disc diameters away from the optic disc. Animals were euthanized 7 days after laser treatment to quantify CNV lesions by multiphoton microscopy.

### CNV lesion assessment.

Enucleated eyes were fixed in 4% PFA. CNV lesions on retinal-choroidal flatmounts were stained for biotin-labeled isolectin B4 (1:50; MilliporeSigma) and Alexa Fluor Streptavidin 568 (1:500; Invitrogen) and imaged using an upright Leica TCS SP8 multiphoton microscope (HC PL Fluotar 20×/0.50 dry lens; Leica Microsystems). Isolectin stained lesions were excited at a wavelength of 830 nm using a Mai Tai DeepSee laser (Spectra Physics) and fluorescence captured using a nondescanned detector. Serial Z-stacks were taken up to a depth of 300 μm at 1.5-μm intervals, allowing images to be collected spanning the full thickness of the CNV lesions. Images were acquired and volume rendered for presentation using Leica LAS-X software. For quantification, images were imported into IMARIS Bitplane 8.4.2 (Bitplane AG), and the total volume of the CNV lesion and total volume of the burn were measured. Data are presented, and were analyzed, as the lesion volume expressed as a percentage of the total burn volume.

### Gross retinal morphology and vascular network analysis.

To assess gross retinal morphology in WT and gene-KO mice (11 months old), eyes were enucleated, prefixed with 10% formalin for 10–30 minutes, and either placed in 10% formalin overnight or dissected to remove the front half of the eyeball, followed by overnight incubation in 10 % formalin. Eyes were then dehydrated and saturated with paraffin (Leica TP1020 tissue processor) and embedded in paraffin blocks using a Leica Arcadia system. Blocks were cut at 5-μm thickness on a microtome (Leica RM2235), floated onto slides, and stained using a Bio-Optica automated slide stainer. H&E-stained slides were imaged on a Leica DMi8 microscope.

Vascular network analysis was performed using isolectin B4–stained retinal flatmounts from adult WT and gene-KO mice (3–6 months old). Retinas were isolated and the vasculature fluorescently stained with biotin-labeled isolectin B4 (1:50; MilliporeSigma) and Alexa Fluor Streptavidin 568 (1:500; Invitrogen). Flat-mounted retinas were imaged using a Leica SP8 resonance scanning confocal microscope (HC PL FLUOTAR 10x 0.3 dry lens; Leica Microsystems). The tile and auto-stitch functions of the microscope were used to produce an image of the entire superficial vascular plexus of the retina. Total vessel length, branching index (number of junctions per mm^2^), and lacunarity were quantified using AngioTool software (76).

### Statistics.

Most statistical analyses were carried out on the raw data using Prism V5.03 (GraphPad Software). Percentage data obtained from the OIR and CNV experiments were arcsine transformed prior to analysis. All datasets were tested to verify that they fulfilled the requirements for a normal distribution (D’Agostino and Pearson omnibus normality test). One-way ANOVA was used followed by Bonferonni post-hoc tests to compare differences among multiple groups. Two-tailed Students *t* test was used to compare data between two groups when appropriate. Cell viability data were analyzed using the χ^2^ test. Data from the phospho-kinase arrays were analyzed by ANOVA using the web-based 1-way NIA array analysis tool (77). This enabled significant changes in the phosphorylation status of individual kinases across treatment groups to be detected, while correcting for multiple comparisons using the FDR method of Benjamini and Hochberg (78). In all experiments, a *P* value of 0.05 was considered statistically significant.

### Study approval.

All animal procedures were approved by Queen’s University of Belfast Animal Welfare and Ethical Review Body (AWERB) and authorized under the UK Animals (Scientific Procedures) Act 1986. Animal use conformed to the standards in the Association for Research in Vision and Ophthalmology (ARVO) Statement for the Use of Animals in Ophthalmic and Vision Research and with European Directive 210/63/EU.

## Author contributions

SA, AWS, JGM, and TMC conceived and designed the experiments. SA, SB, COL, PC, IM, MOH, PB, HM, and DPB performed the experiments. SA, SB, COL, IM, JAF, MOH, PB, HM, DPB, and TMC analyzed the data. SA, AWS, JGM, and TMC wrote the paper. JAF wrote the OIR macro and the OIR macro user guide. All authors read and approved the final manuscript for submission.

## Supplementary Material

Supplemental data

Supplemental data 2

## Version 1 02/05/2019

In-Press Preview

## Version 2 03/21/2019

Electronic publication
