# Supplementary material for: CAMKII as a therapeutic target for growth factor–induced retinal and choroidal neovascularization
Source: JCI Insight. 2019 Mar 21;4(6):e122442. doi: 10.1172/jci.insight.122442 (PMC6482993; doi:10.1172/jci.insight.122442)
Supplement: Supplemental data [file jciinsight-4-122442-s095.pdf]

A

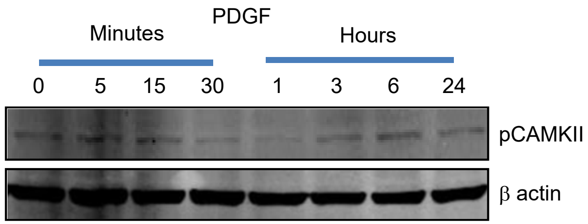

B

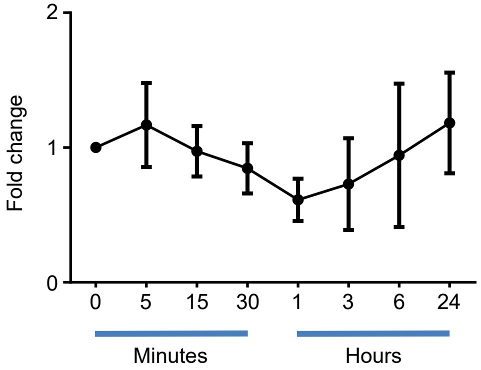

Supplementary Figure 1. Effects of PDGF on Ca^{2+}/calmodulin-dependent kinase II (CAMKII) phosphorylation. A, B. Representative western blot gels (A) and summary data (B) showing that PDGF failed to trigger CAMKII phosphorylation in human retinal microvascular endothelial cells. Data information: data represent mean ± SEM; n=4 biological replicates.

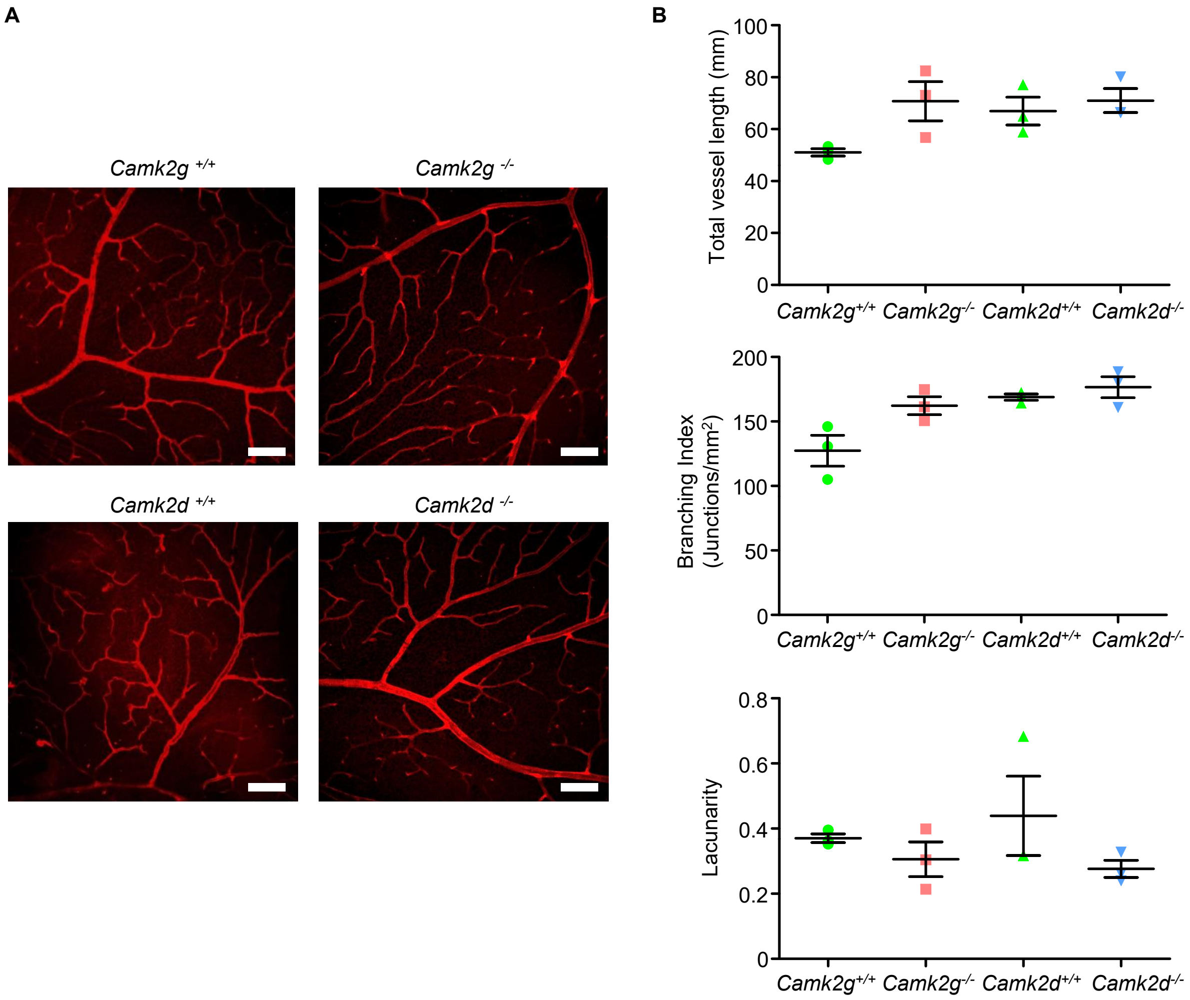

Supplementary Figure 2. Retinal vascular network analysis in adult Ca^{2+}/calmodulin-dependent kinase II (CAMKII) γ and δ wild-type and knockout mice. A. Representative regions of isolectin B4-stained retinas from CAMKIIγ and δ wild-type and knockout mice imaged at the level of the superficial vascular plexus. Scale bars = 100μm. B. Quantification of total vessel length, branching index (number of junctions per mm^2) and lacunarity among the different groups of animals calculated using AngioTool software. Data information: data represent mean ± SEM; n=3 mice per group.

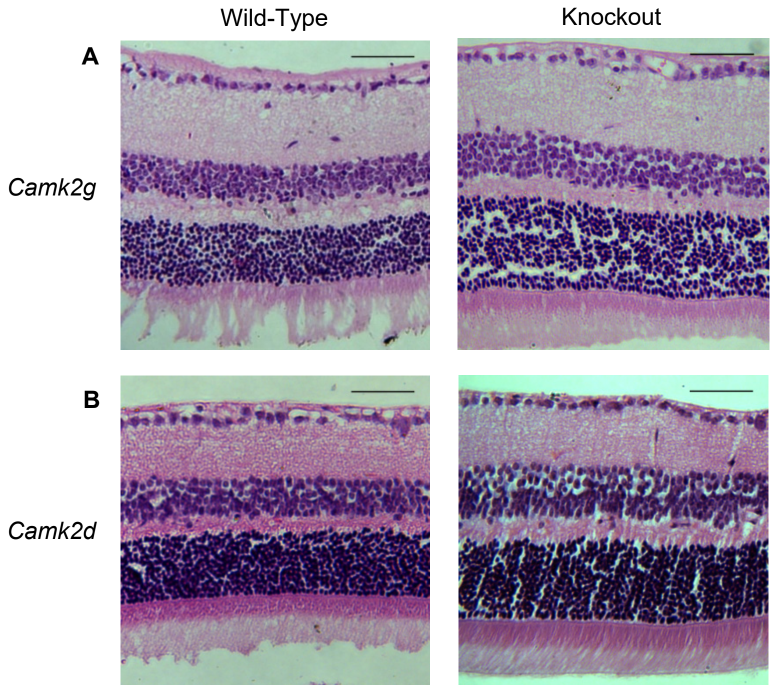

Supplementary Figure 3. Gross retinal morphology in adult Ca^{2+}/calmodulin-dependent kinase II (CAMKII) γ and δ wild-type and knockout mice. A, B. Eyecups were fixed, embedded in paraffin, cut at 5μm and stained with haematoxylin and eosin to visualise the gross structural morphology of the retina. Retinal morphology appeared normal in both the wild-type and knockout mice. Data information: images are representative of retinal sections from 6 animals in each group. Scale bars = 50 μm.

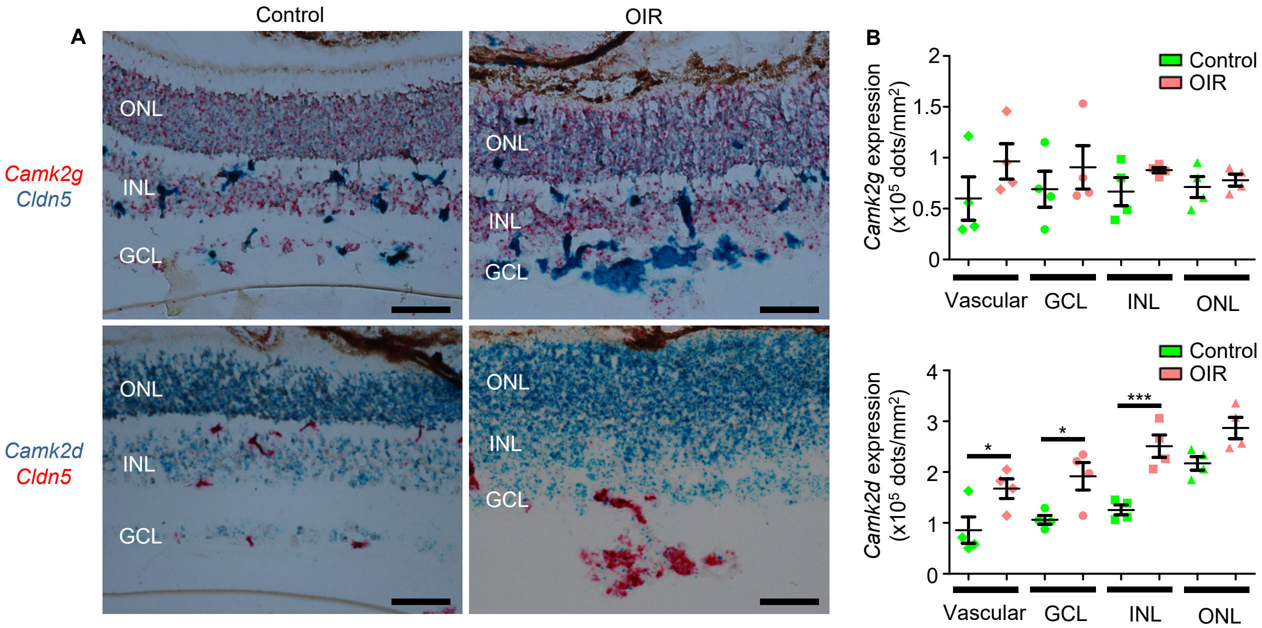

Supplementary Figure 4. Cellular localisation of Ca^{2+}/calmodulin-dependent kinase II (CAMKII) γ and δ mRNA in the normal and ischemic retina. A. Representative RNAscope in situ hybridization images of *Camk2g* (top panels, red) and *Camk2d* (bottom panels, blue) in retinal sections from P15 control (normoxic) and oxygen-induced retinopathy mice. Retinal sections were co-labelled using RNAscope probes against *Cldn5* (top panels, blue; bottom panels, red) to mark the vascular endothelial cells. GCL, ganglion cell layer; INL, inner nuclear layer; ONL, outer nuclear layer. Scale bars = 50µm. B. Scatter dot plot showing *Camk2g* (top panel) and *Camk2d* (bottom panel) mRNA expression in selected retinal regions presented as the average number of RNAscope dots per mm^2 of tissue. Data information: data represent mean ± SEM; * $p<0.05$, *** $p<0.001$ based on ANOVA. n=4 mice per group.

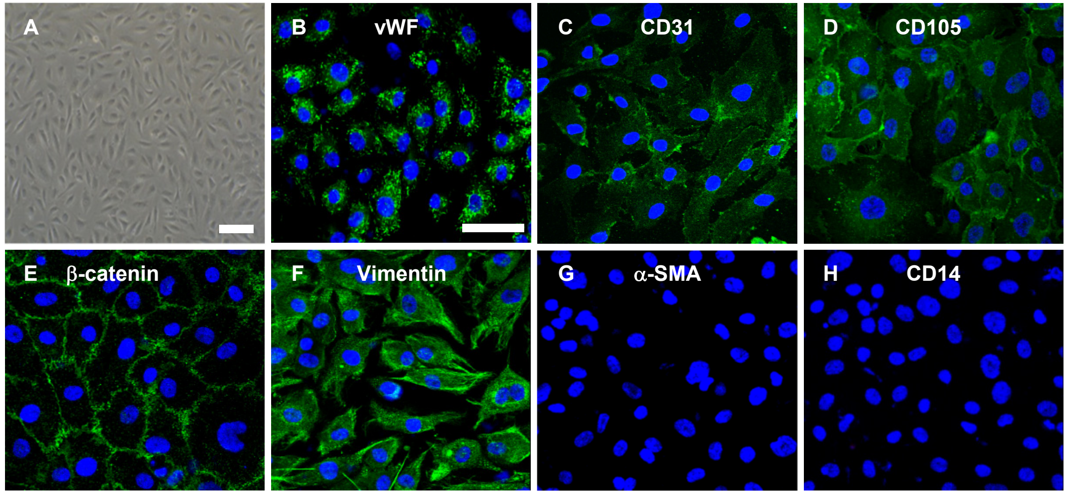

Supplementary Figure 5. Confirmation of endothelial characteristics of primary human retinal microvascular endothelial cells (HRMECs). A. HRMECs display the typical morphology of cultured endothelial cells when viewed under phase contrast microscopy. B-F. Immunolabelling studies confirmed that HRMECs represent a homogenous population of cells with endothelial characteristics. Granular cytoplasmic staining for von Willebrand factor (vWF) was observed (B). CD31 (PECAM-1) staining was seen primarily on borders between endothelial cells (C) and proliferating HRMECs exhibited cytoplasmic CD105 (endoglin) staining (D). They also formed adherens junctions as indicated by membrane localisation of β-catenin (E) and were positive for the intermediate filament cytoskeletal marker vimentin (F). G, H. Alpha-smooth muscle actin (α-SMA; G) and CD14 (H) were absent, suggesting no contamination by smooth muscle cells or macrophages. Data information: images of representative of 3 biological replicates. Scale bars: 100μm.

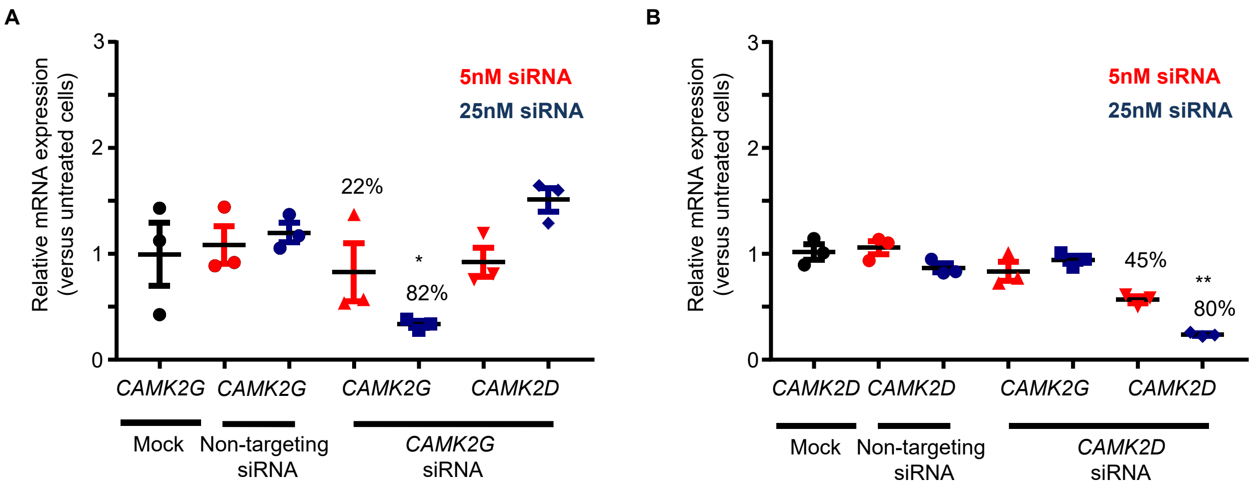

Supplementary Figure 6. Silencing of CAMKIIγ and δ isoforms in human retinal microvascular endothelial cells (HRMECs). A, B. HRMECs were either mock transfected or transfected with 5 or 25nM non-targeting, *CAMK2G* (A) or *CAMK2D* (B) siRNAs. *CAMK2G* and *CAMK2D* mRNA transcripts were quantified 24 hours later by qPCR (normalised to *GAPDH*) and data expressed relative to untreated (control) cells. 25nM siRNA was required for effective knockdown of *CAMK2G* and *CAMK2D* isoforms. Silencing one isoform had no significant effect on the expression of the other. Data information: data represent mean ± SEM; * $p<0.05$, ** $p<0.01$ based on ANOVA. n=3 biological and 3 technical replicates.

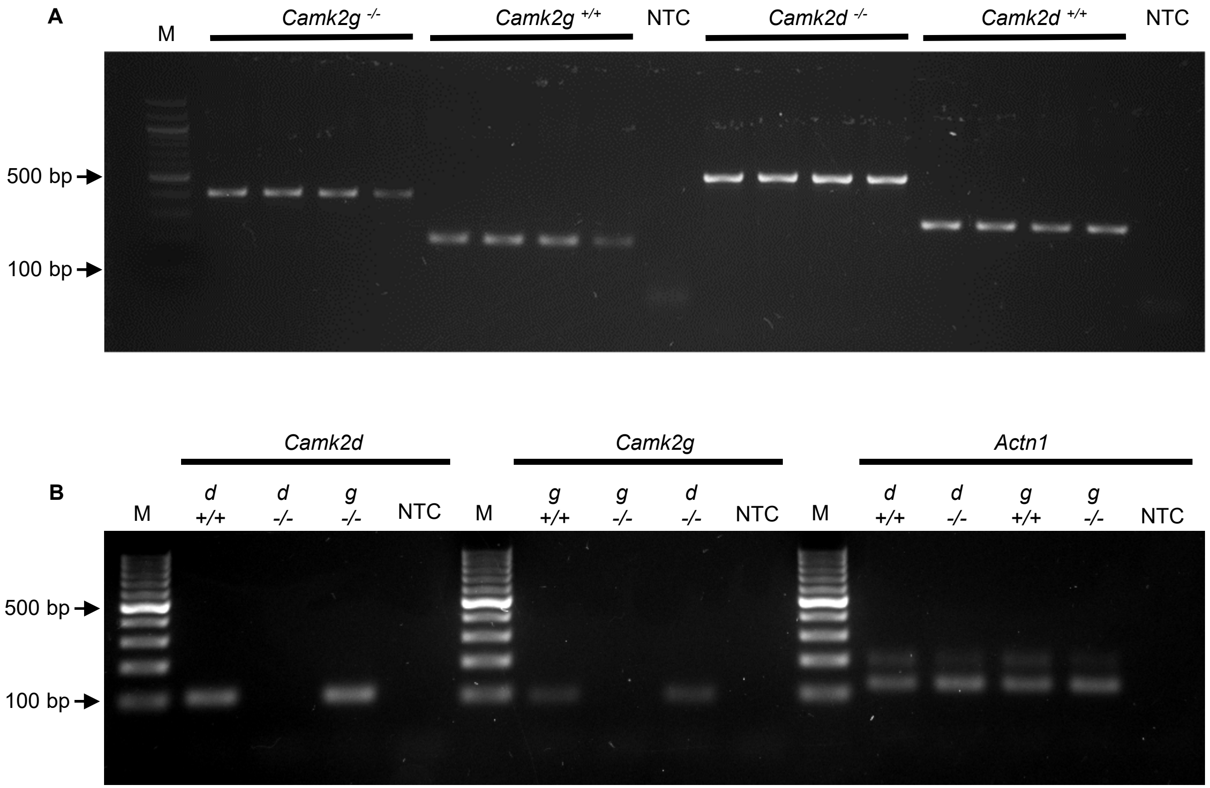

Supplementary Figure 7. Genotyping and validation of retinal gene deletion in Ca^{2+}/calmodulin-dependent kinase II (CAMKII) γ and δ knockout mice. A. Representative genotyping gel of DNA from ear biopsies of CAMKIIγ and δ homozygous knockout and wild-type mice. Each lane represents an individual animal. The lengths of the PCR products were as expected based on the primer sets used (see Methods). B. RT-PCR analysis of total RNA extracted from the retinas of CAMKIIγ and δ wild-type and homozygous knockout mice. CAMKIIγ and δ bands were absent in the respective gene knockout mice. Expression of *Actn1* (Actinin-1) was used as a positive control for the PCR reaction. In both gels, “M” refers to the molecular weight marker lane and “NTC” to no template controls, where PCR-grade water was substituted for the template.

Kinase	Site	VEGF	VEGF+KN92	VEGF+KN93	VEGF vs Control	VEGF vs VEGF+KN92	VEGF vs VEGF+KN93
		Fold change	Fold change	Fold change	P	P	P
**Akt 1/2/3**	S473	1.68	1.79	1.39	P < 0.001	P > 0.05	P < 0.001
**Akt 1/2/3**	T308	0.91	0.89	1.04	P > 0.05	P > 0.05	P > 0.05
**AMPKα1**	T183	1.37	1.49	1.31	P < 0.001	P > 0.05	P > 0.05
**AMPKα2**	T172	1.30	1.32	1.27	P > 0.05	P > 0.05	P > 0.05
**Chk-2**	T68	1.58	1.33	1.24	P < 0.001	P < 0.01	P < 0.01
**c-Jun**	S63	0.84	0.81	1.00	P > 0.05	P > 0.05	P > 0.05
**CREB**	S133	1.58	1.25	1.34	P < 0.01	P > 0.05	P > 0.05
**EGF R**	Y1086	2.08	1.76	1.44	P < 0.05	P > 0.05	P > 0.05
**eNOS**	S1177	0.88	0.84	1.15	P > 0.05	P > 0.05	P > 0.05
**ERK1/2**	T202/Y204,T185/Y187	1.31	1.37	1.23	P > 0.05	P > 0.05	P > 0.05
**FAK**	Y397	1.57	1.49	1.17	P < 0.001	P > 0.05	P < 0.01
**Fgr**	Y412	1.46	1.42	1.17	P > 0.05	P > 0.05	P > 0.05
**Fyn**	Y420	1.29	1.25	1.10	P > 0.05	P > 0.05	P > 0.05
**GSK-3α/β**	S21/S9	1.20	1.17	1.21	P > 0.05	P > 0.05	P > 0.05
**Hck**	Y411	1.35	1.28	1.08	P > 0.05	P > 0.05	P > 0.05
**HSP27**	S78/S82	1.64	1.57	1.34	P < 0.01	P > 0.05	P > 0.05
**HSP60**	-	0.90	1.11	0.97	P > 0.05	P > 0.05	P > 0.05
**JNK 1/2/3**	T183/Y185, T221/Y223	1.44	1.46	1.13	P < 0.001	P > 0.05	P < 0.01
**Lck**	Y394	1.41	1.26	1.14	P < 0.05	P > 0.05	P > 0.05
**Lyn**	Y397	1.26	1.11	1.10	P > 0.05	P > 0.05	P > 0.05
**MSK1/2**	S376/S360	1.44	1.41	1.18	P > 0.05	P > 0.05	P > 0.05
**p27**	T198	1.08	1.14	1.55	P > 0.05	P > 0.05	P > 0.05
**p38α**	T180/Y182	1.64	1.66	1.64	P < 0.001	P > 0.05	P > 0.05
**p53**	S392	0.95	0.93	1.02	P > 0.05	P > 0.05	P > 0.05
**p53**	S15	0.89	0.93	1.03	P > 0.05	P > 0.05	P > 0.05
**p53**	S46	0.89	0.93	0.99	P > 0.05	P > 0.05	P > 0.05
**p70 S6 Kinase**	T389	1.02	0.93	1.39	P > 0.05	P > 0.05	P > 0.05
**p70 S6 Kinase**	T421/S424	1.01	0.97	1.17	P > 0.05	P > 0.05	P > 0.05
**PDGF Rβ**	Y751	1.51	1.88	1.32	P < 0.05	P > 0.05	P > 0.05
**PLC-γ1**	Y783	0.85	0.83	1.07	P > 0.05	P > 0.05	P > 0.05
**PRAS40**	T246	1.08	1.09	1.13	P > 0.05	P > 0.05	P > 0.05
**PYK2**	Y402	0.95	0.98	0.87	P > 0.05	P > 0.05	P > 0.05
**RSK 1/2/3**	S380/S386/S377	0.92	0.92	1.08	P > 0.05	P > 0.05	P > 0.05
**Src**	Y419	1.41	1.32	1.13	P < 0.01	P > 0.05	P < 0.05
**STAT2**	Y689	1.31	1.35	1.31	P > 0.05	P > 0.05	P > 0.05
**STAT3**	Y705	1.62	1.41	2.23	P > 0.05	P > 0.05	P > 0.05
**STAT3**	S727	1.50	1.31	1.82	P > 0.05	P > 0.05	P > 0.05
**STAT5a**	Y694	1.33	1.37	1.30	P > 0.05	P > 0.05	P > 0.05
**STAT5a/b**	Y694/Y699	1.11	1.25	1.17	P > 0.05	P > 0.05	P > 0.05
**STAT5b**	Y699	1.14	1.30	1.21	P > 0.05	P > 0.05	P > 0.05
**STAT6**	Y641	1.19	1.25	1.19	P > 0.05	P > 0.05	P > 0.05
**TOR**	S2448	1.45	1.28	1.11	P > 0.05	P > 0.05	P > 0.05
**WNK1**	T60	1.03	1.17	1.34	P > 0.05	P > 0.05	P > 0.05
**Yes**	Y426	1.45	1.32	1.18	P < 0.01	P > 0.05	P < 0.05
**β-Catenin**	-	1.56	1.54	1.22	P < 0.001	P > 0.05	P < 0.05

Supplementary Table 1. Summary of phospho-kinase array results. The R&D Systems Human Phospho-Kinase Antibody Array detects the relative site specific phosphorylation of 43 kinases and 2 related total proteins (HSP60 and β-catenin). The normalised intensity for each antibody for human retinal microvascular endothelial cells exposed to VEGF in the absence or presence of KN93 or KN92 is presented as a fold change compared to untreated (control) cells. Statistical comparisons were performed using NIA Array Analysis software.
