# Supplementary material for: CAMKII as a therapeutic target for growth factor–induced retinal and choroidal neovascularization
Source: JCI Insight. 2019 Mar 21;4(6):e122442. doi: 10.1172/jci.insight.122442 (PMC6482993; doi:10.1172/jci.insight.122442)
Supplement: Supplemental data 2 [file jciinsight-4-122442-s096.zip › OIR Select/User manual/OIR Select - User Manual.pdf]

***OIR Select*: A program for the quantification of vascular, avascular and neovascular regions**

José A. Fernández

Developed at:

Centre for Experimental Medicine, The Queen's University of Belfast, UK

Contents

1. Introduction

2. Getting started

    2.1. Main idea behind the algorithm

    2.2. Running the *OIR Select* macro

3. Working example: Analysis of a retina

4. Other parameters of interest

1. Introduction

OIR Select is an ImageJ macro created to assist researchers in the task of estimating and measuring vascular, avascular and neovascular areas in flatmounted retinas used within the oxygen-induced retinopathy (OIR) model. The algorithm uses the thresholding methods to independently analyse different regions of the same retina. Relative measurements of the different regions of interest are provided as percentages of the whole retina. The program is easy to run from the ImageJ macro window and guides the user through a series of steps until the retina is fully analysed.

This macro is made freely available, open-source and has been distributed under the terms of the GNU General Public License as published by the Free Software Foundation, either version 3 of the License, or (at your option) any later version.

2. Getting started

2.1. Main idea behind the algorithm

The program requires a retinal flatmount image as input with the vasculature labelled (Fig. 1 A). Four threshold values are sequentially asked from the user. Each value is used by the program to mark a different region in the retina (i.e. whole retina, peripheral and central vascular, and neovascular regions). The Huang (whole retina) and Otsu (vascular, avascular and neovascular) thresholding methods are used for this.

Two parameters are initially used to determine (a) the smoothness of the detection of the whole retina, and (b) a minimal central region in the retina where no neovascular regions will be found (see section 3 below for further explanation). Once the process starts, the user independently selects a threshold for each of the four regions indicated above. Setting a threshold too high will miss important regions of interest, whereas setting it too low will falsely misclassify regions. To assist with the selection of the threshold value, a live black-and-white mask window is presented at each stage for the user to visually check that the white template (set by the threshold) covers the specific regions of interest (Fig. 1 B–E).

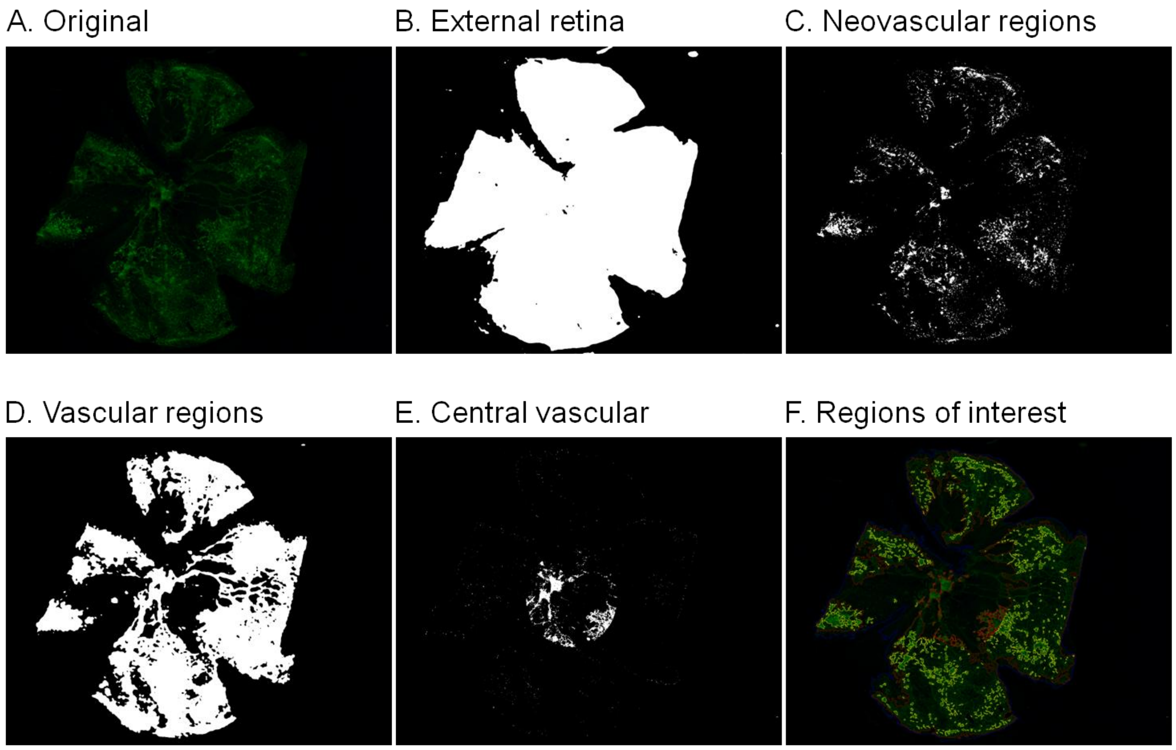

Figure 1. Computer-aided quantification of regions of interest within the retina. (A) Original image of a flatmounted retina from OIR experiment fluorescently stained with biotin-labelled isolectin B4 and Alexa-488 streptavidin. (B–E) White regions indicate detected areas determined by different selected threshold values. (F) Final image of the regions of interest determined by the thresholds in different colours (dark blue for whole retina, red for vascular/avascular regions, and yellow for neovascular regions).

Once a threshold is selected, this value is internally recorded and the next selection opens up until all four regions have been marked. Finally, the program presents the full retina to the user with the different regions marked with lines of different colours (Fig. 1 F). The program also quantifies the vascular (central plus peripheral), avascular and neovascular regions and provides these areas as percentages of the overall retina. As noticed in Figure 1, the analysis of the central vasculature is separated from the rest of the analysis. This is done to avoid the computer confusing these regions with neovasculature (see section 3 below). A table of results is also provided, containing different measurements taken for each of the regions identified for posterior analysis.

2.2. Running the *OIR Select* macro

To run *OIR Select*, FIJI (FIJI Is Just ImageJ) is needed. A version equal or higher than 1.49h should be first installed in the computer. This can be done by opening the FIJI website:

http://fiji.sc/Fiji

and following the installation instructions after clicking on Download FIJI now.

After downloading FIJI, the application can be run directly from the folder obtained by double clicking on the FIJI application icon. When this is done, a small menu opens:

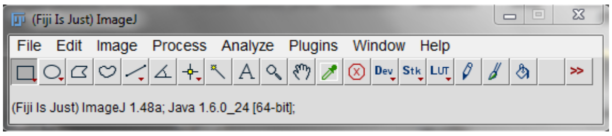

Figure 2. Running FIJI application on the computer

After installing ImageJ, the version obtained should be the latest available. If this is not the case, the software can be updated by clicking on Help -> Update ImageJ...

To open the *OIR Select* macro, just drag and drop the IJM file provided into the FIJI menu window. A window for developing code will open with the code of the *OIR Select* macro in it:

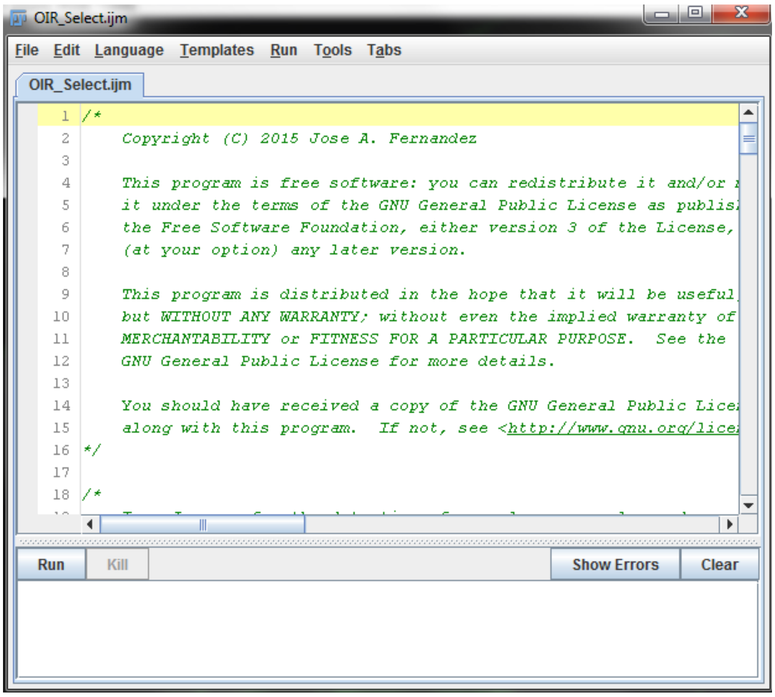

Figure 3. Opening the macro *OIR Select* file in FIJI

This window can be used to see, change and adapt the code, as well as to modify the parameters used for the processing. The code in this macro has been extensively commented to facilitate changes by users.

To run the macro, once the OIR image to be analysed is opened in the Desktop, the user just needs to press the Run button at the bottom left of the window (Fig. 3). The macro then runs and guides the user through the steps needed to carry out the analysis of the retina (see section 3 below for an example).

3. Working example: Analysis of a retina

To analyse a retina, the following steps are needed:

A. Open an image of interest using the ImageJ menu (File -> Open):

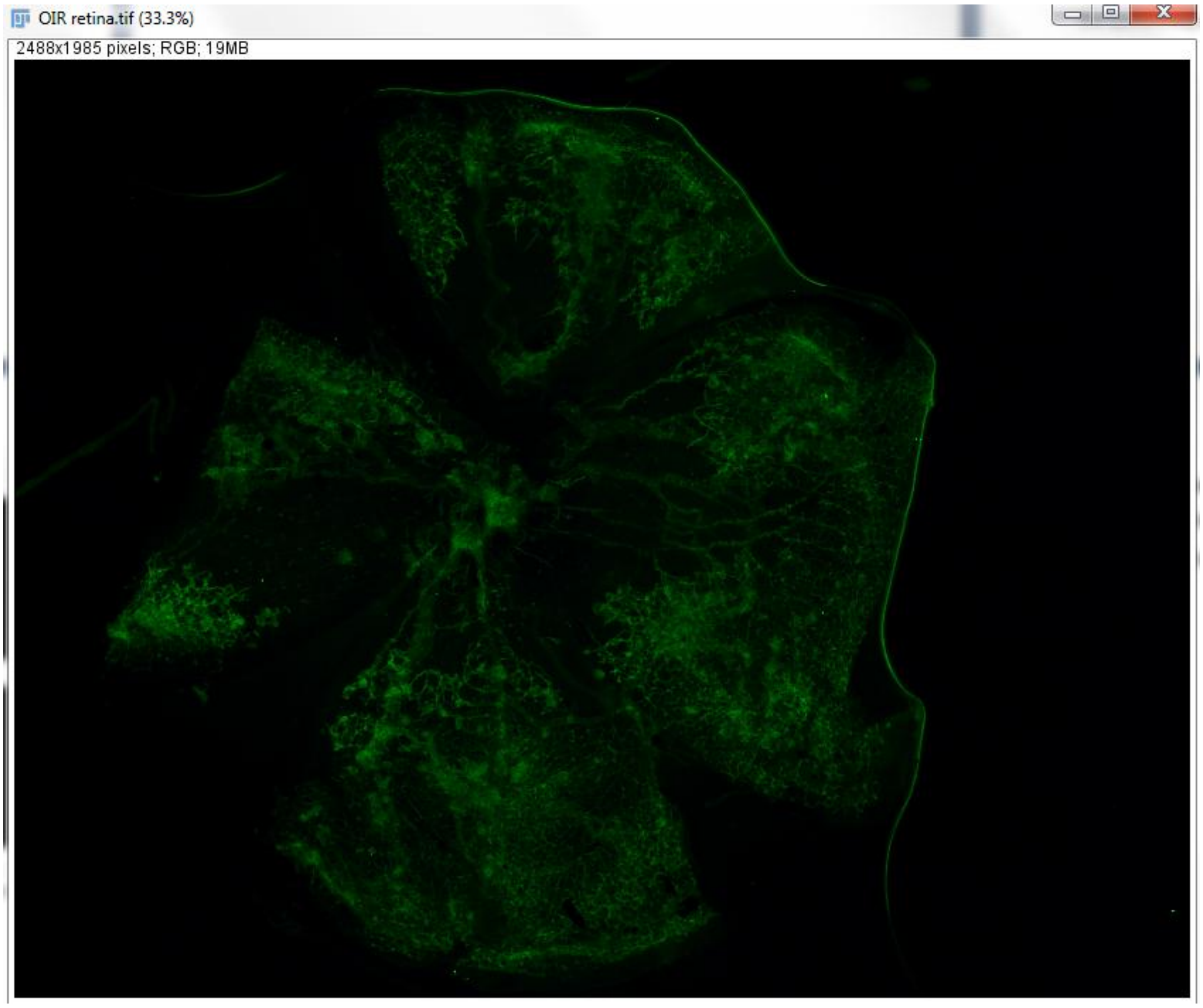

Figure 4. Image of a retina taken from an OIR experiment opened with FIJI

Occasionally, it may be necessary to pre-process the image to remove any artefacts that can be clearly identified by eye. Otherwise, the thresholding methods to be applied below may detect structures that are not really part of the retina. In this

example, we need to use the ImageJ tools to remove the bright line in the external border of the retina (Fig. 4). After this is done, the resulting image is shown below:

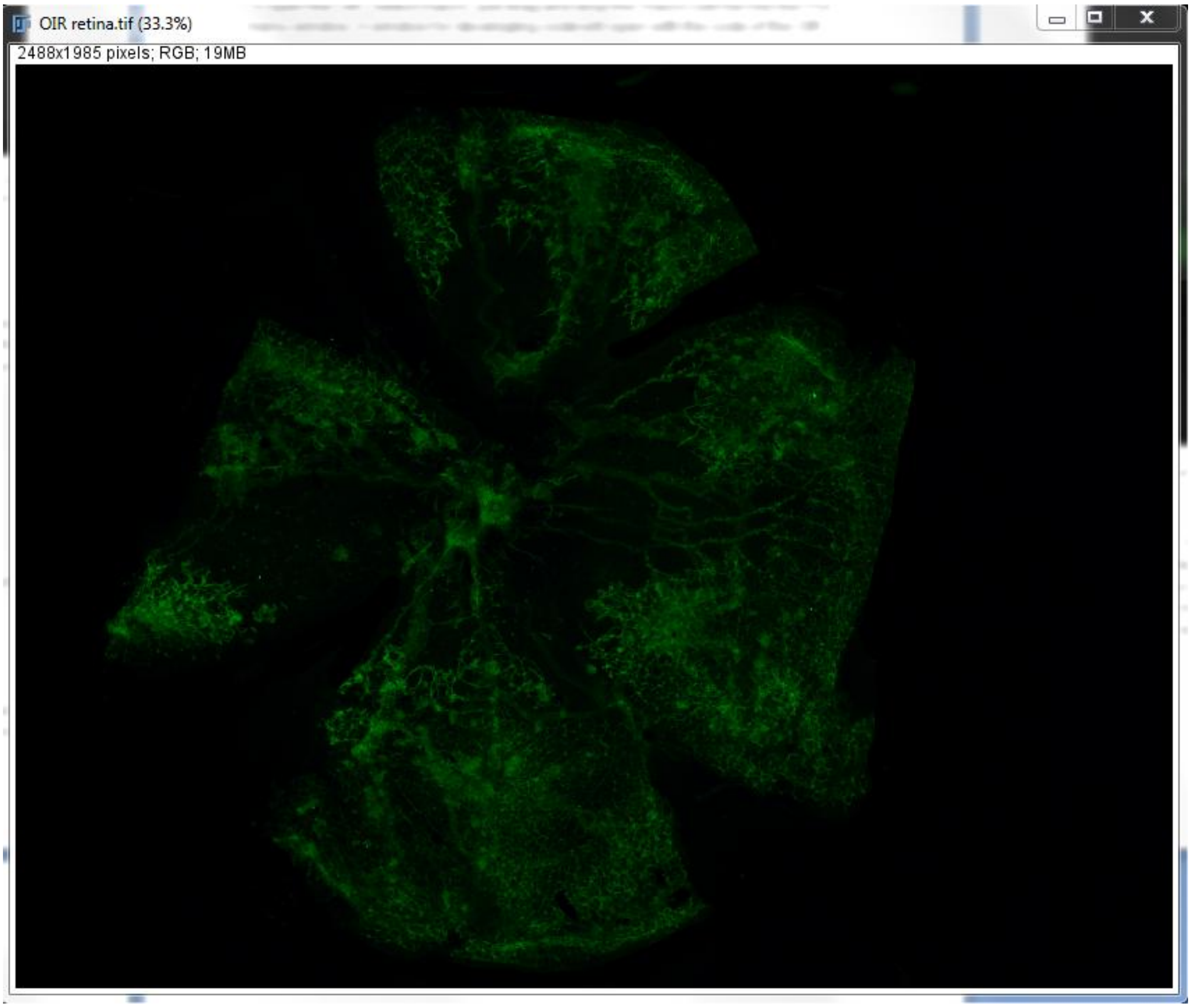

Figure 5. Pre-processed image of the same retina shown in Figure 4

B. Click on Run in the macro window for developers (Fig. 3). A small window is shown for the user to select two parameters:

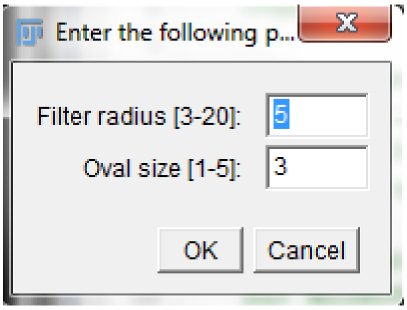

Figure 6. Window for the selection of parameters for analysis

B.1. The first parameter, Filter radius, indicates the amount of smoothing we want to apply to the detection of the different areas. The value of the parameter corresponds to the main parameter in the underlying Gaussian filter applied to the image (i.e. its sigma value). A default sigma value of 5 is provided. Values can range from 3 to 20, representing two extremes in the smoothing being applied. For example, a sigma of 3 would follow the rough edges of the retina more closely:

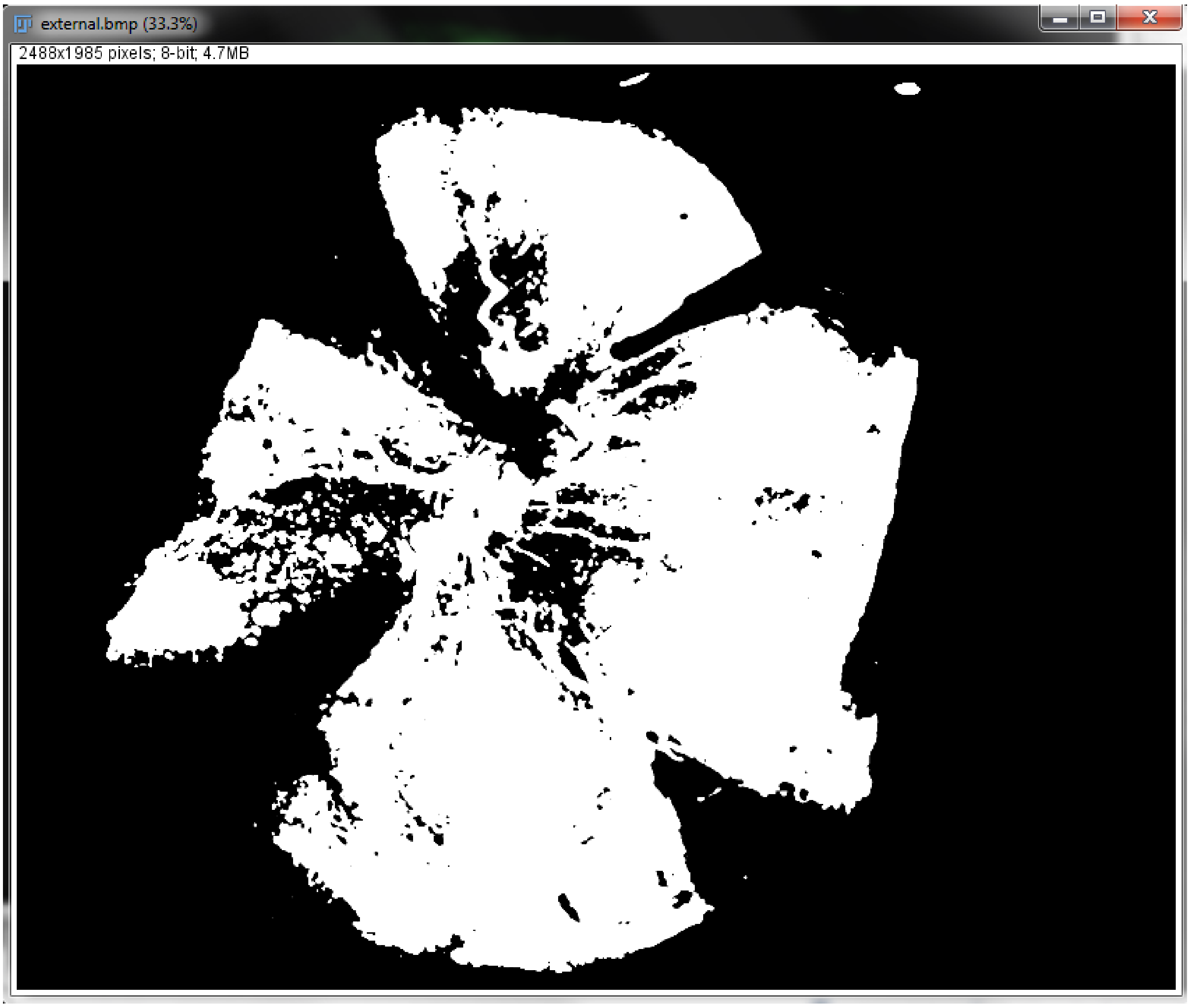

Figure 7. Thresholded retina after applying a Gaussian filter with sigma = 3

whereas a sigma of 20 would substantially smooth the edges:

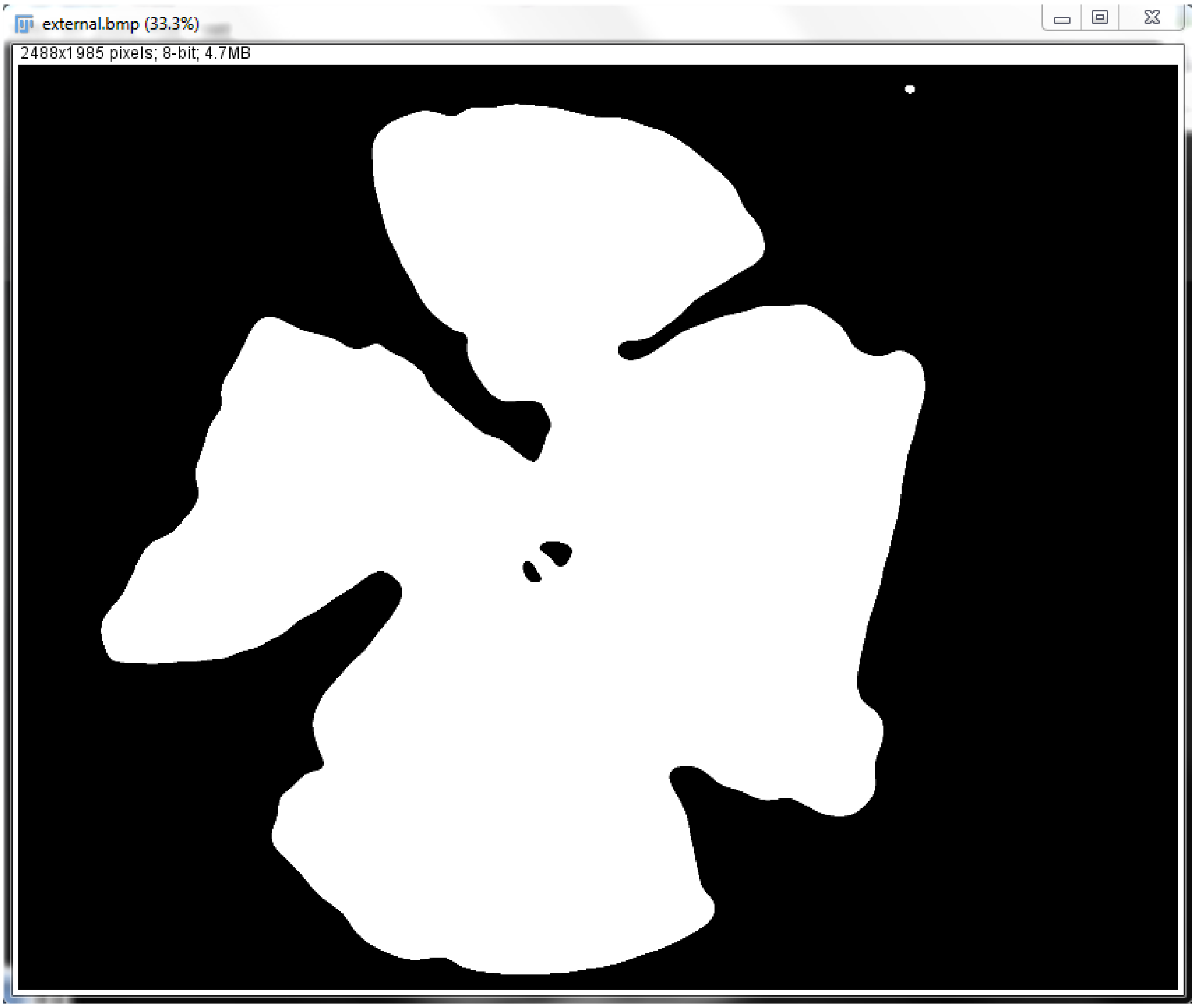

Figure 8. Thresholded retina after applying a Gaussian filter with sigma = 20

The smoothing parameter is applied to the detection of the full retina and of the peripheral vascular areas (but not to the detection of the central vascular or the neovascular areas). Modifying this parameter should not affect dramatically the final analysis. In this particular example, the relative percentages given at the end of the analysis for vascular and avascular areas with the two different sigma values are very similar (relative vascular area is 33% of the total retina for a sigma value of 3, and 32% for a sigma value of 20).

B.2. The second parameter in the window, Oval size, indicates the minimum size of a oval drawn in the centre of the retina surrounding the central vasculature. The

purpose of adding this extra feature to the software is to be able to process the central vasculature independently from the rest of the areas. The reason for this is that generally the central vasculature is very bright and can be confused for neovasculature by the software. By using an oval, we are able to delimit a central area where no neovasculature is to be found.

The size of the oval ranges from 1 (biggest) to 5 (smallest). In this example, an oval size of 1 would practically cover the full retina, indicating that almost no neovascular regions are to be found in the whole retina area (using an oval size of 1 and leaving all other parameters as default gives a value for the neovascular area of 0.26% of the total retina). A size of 5 will select a very small oval, enough just to surround the optic nerve spot and little else. This will most likely overestimate the neovascular areas by picking up bright areas in the centre of the retina that likely do not correspond to neovascular areas. A default value of 3 is usually good enough to cover the central vasculature.

C. Once the two parameters have been entered (or the defaults have been accepted), the program asks the user to select the first threshold to mark the full retina. For this, four different windows pop up to assist the user:

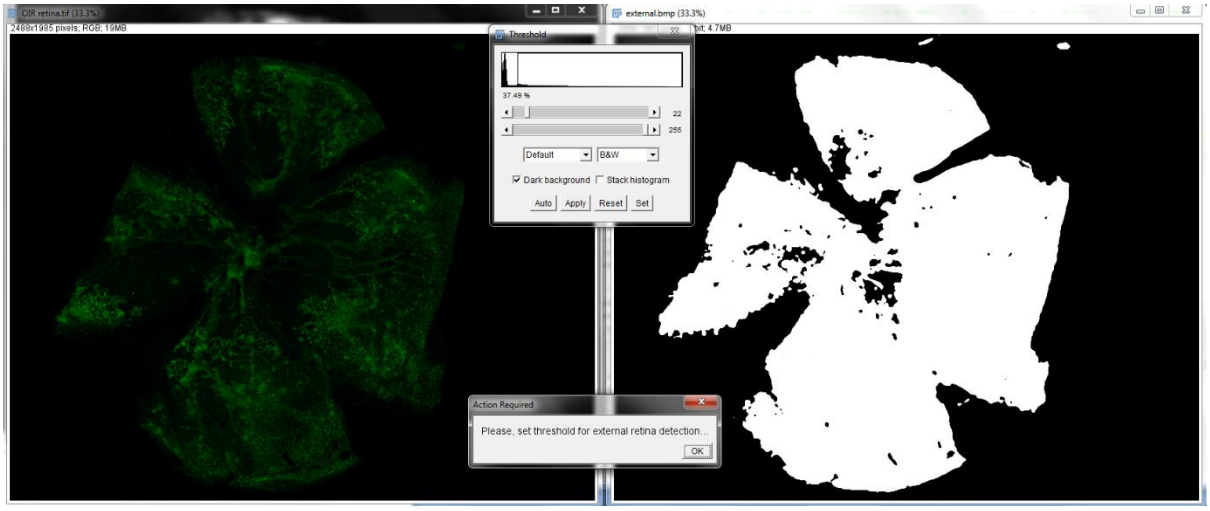

Figure 9. Windows needed to select the first threshold to mark the full retina

Two of the windows contain the original image (Fig. 9, left) and the thresholded image (Fig. 9, right). The other two are a window to modify the thresholding values used (Fig. 9, inset middle top) and a small window to confirm this step when finished (Fig. 9, inset middle bottom). The idea here is to check the thresholded image on the right to make sure that the white template covers the regions we want to detect in the left. The white template can be changed by scrolling left or right in the thresholding window (Fig. 9, inset middle top). Once the white regions cover the full retina, we

press OK in the small inset window at the bottom (Fig. 9, inset middle bottom) to continue with the analysis. It is important to make sure at this point that there is one main white region detected that contains most of the retinal area so that the program can identify with certainty where the full retina is. Notice that detecting the wrong area at this point, or detecting only part of it, will have an impact on all the relative measurements provided at the end of the analysis.

D. Once we press OK, the program asks the user to select the second threshold to mark the vascular areas. Again, four windows pop up to assist the user with this:

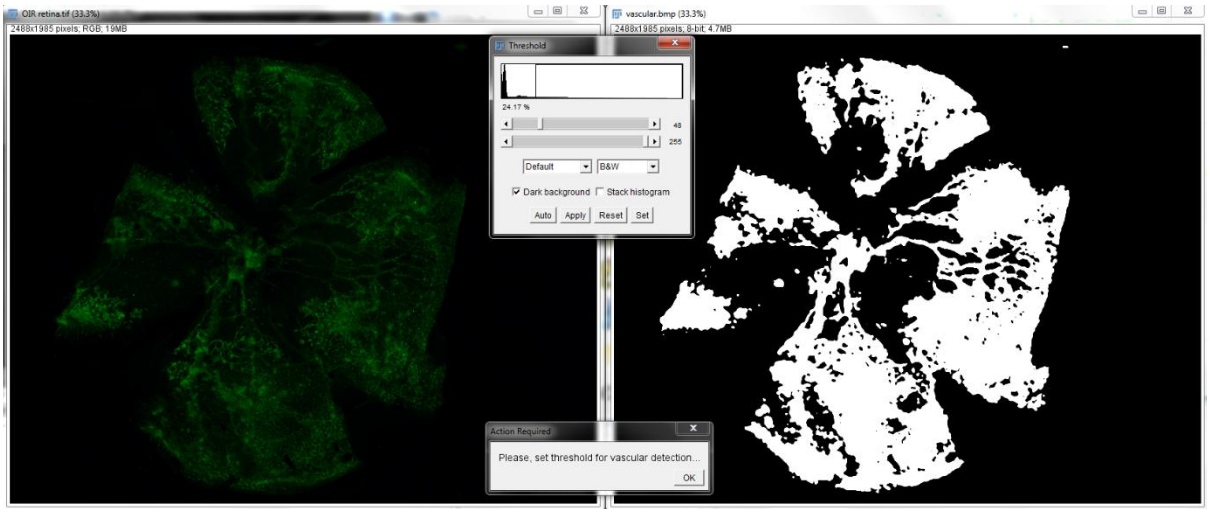

Figure 10. Windows needed to select the second threshold to mark the vascular regions

The process is exactly the same as in (C) above, with the only difference that the user now has to check that the white template (Fig. 10, right window) covers the vascular regions as identified by eye in the left window (Fig. 10, left window).

E. In the next step the program asks the user to select the third threshold to mark the central vasculature (region found within the oval, explained in B.2 above). The usual four windows pop up (Fig. 11):

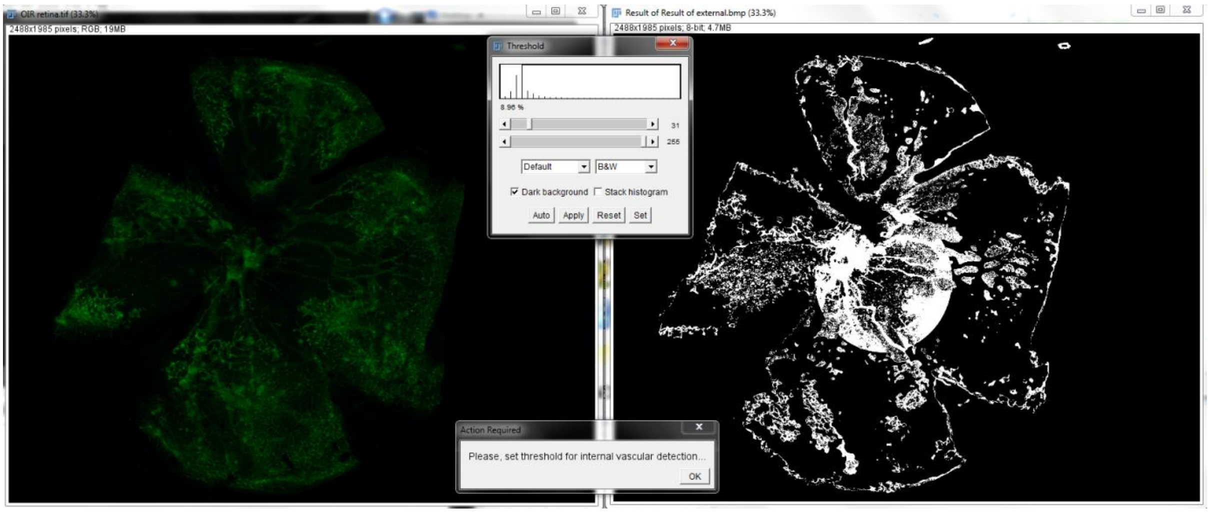

Figure 11. Windows needed to select the third threshold to mark the central vascular regions

The user will generally need to adjust the threshold value to allow the white template to cover the central bright regions of the retina where we are certain no neovasculature is to be found (Fig. 12):

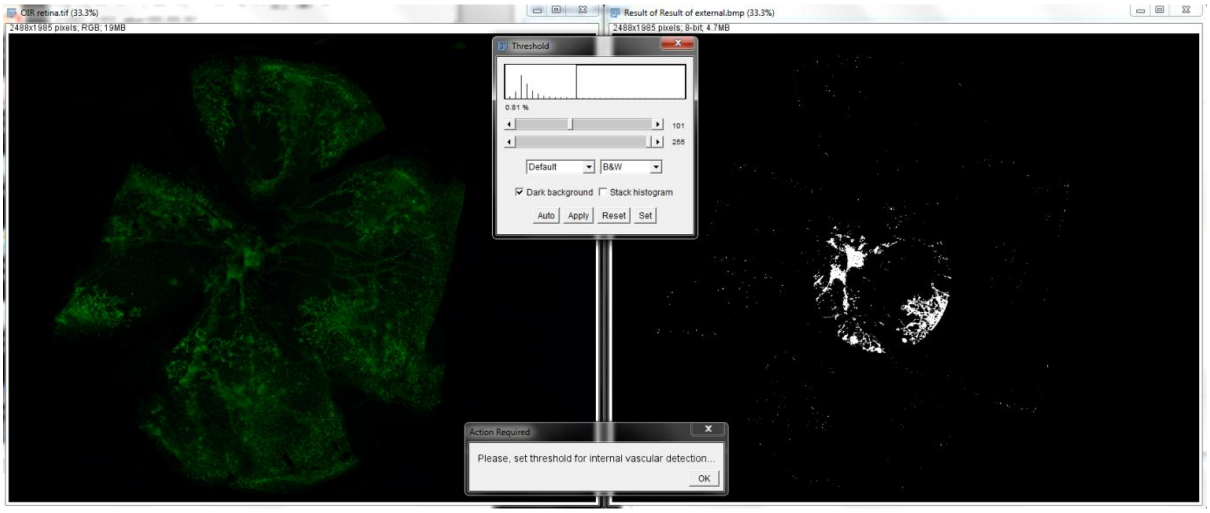

Figure 12. Windows needed to select the third threshold to mark the central vascular regions. The threshold has been adjusted in this case to cover only the central regions where we are certain no neovasculature is found.

F. Finally, the program asks the user to select the fourth and final threshold to mark the neovasculature. Here, again, we have to select the threshold for which the white template best covers the neovascular regions we can identify by eye (Fig. 13):

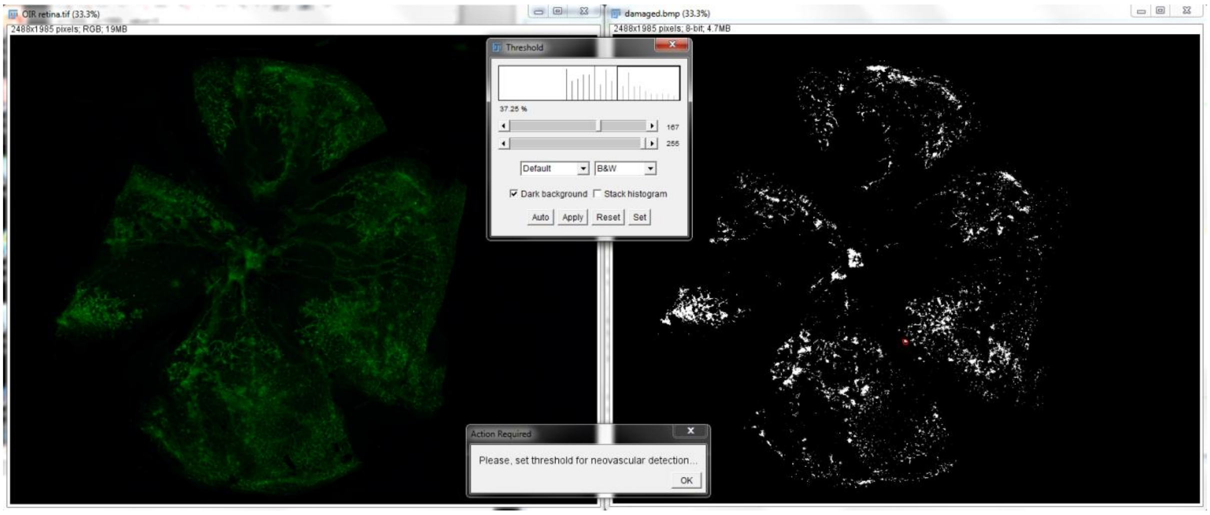

Figure 13. Windows needed to select the final threshold to mark the neovascular regions

G. At the end of the analysis, the program provides a few windows with the following measurements (Fig. 14):

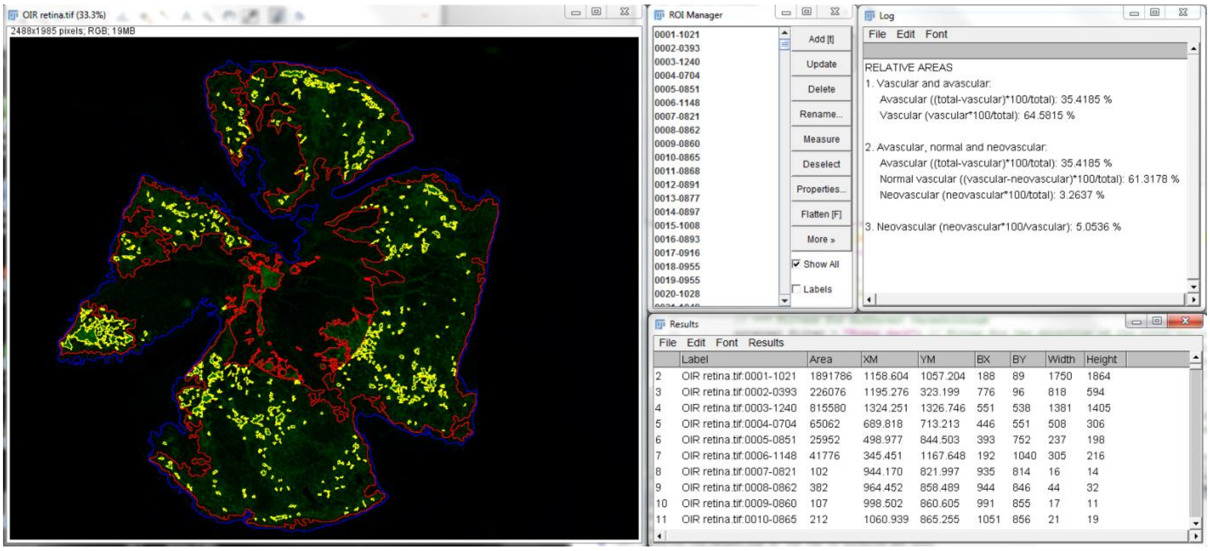

Figure 14. Windows with resulting measurements and areas of interest

G.1. The left window (Fig. 14, left) shows the full retina with an overlay of three lines of different colours delineating the full retina (blue line), vascular areas (red line) and neovascular areas (yellow line). The width of these lines can be changed by modifying one of the parameters in the macro (line 53 of the code). ImageJ allows the user to flatten this overlay (Image -> Overlay -> Flatten) to draw these lines directly on the image. The image can then be saved for posterior use.

G.2. The ROI Manager window (Fig. 14, middle top) contains all the regions of interest detected. This window allows the user to select individual regions from the main image and measure them individually. It also allows the user to control the formatting of the lines in the main image (e.g. line width, colours, etc).

G.3. The Log window (Fig. 14, right top) gives the relative vascular, avascular and neovascular areas as percentages of the total retina. The line at the bottom of the Log also provides a measurement of the neovascular areas relative to the vascular ones. The formulas used to do these calculations are also indicated.

G.4. Finally, the Results window (Fig. 14, right bottom) provides several measurements for each of the areas detected. The kinds of measurements that can be obtained are set using an ImageJ menu (Analyze -> Set Measurements...).

4. Other parameters of interest

A couple of parameters have been added to the software that may be of interest to the user.

4.1. damaged_removed (line 52 of the code): This parameter is used to set the minimum size that the neovascular regions need to be to be detected. The figure below shows two examples of detections with values of 0 (i.e. detect all regions; Fig. 15, left) and 200 (i.e. detect regions with size above 200; Fig. 15, right):

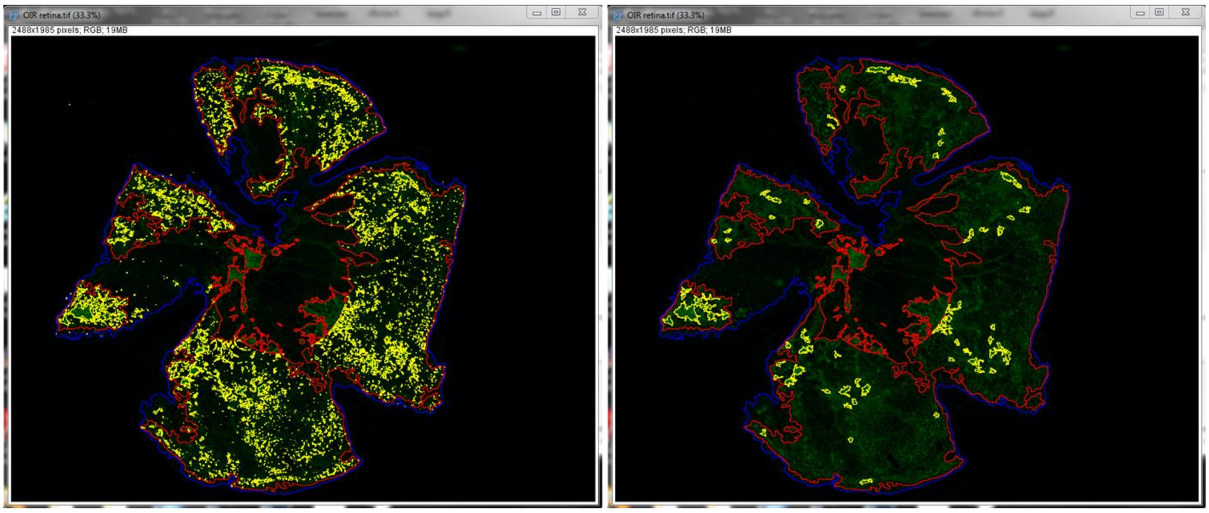

Figure 15. Two examples of neovascular regions detected with different values in the parameter damaged_removed. Full retina and vascular regions are the same in both images

Although the neovascular regions detected look very different in the two images, this difference is not so big in terms of percentages relative to the total retina (4.5% in the image on the left, and 2% in the image on the right). However, the difference in the number of regions detected is very big indeed (5619 regions in the left as opposed to 98 regions in the right). The reason for this difference is that a value of 0 forces the software to report every single region detected as long as they are 1 pixel in size or bigger. Reporting such a big number of regions will make it very difficult to search for individual regions and may complicate subsequent analysis. On the other hand, selecting too big a value for this parameter may miss small neovascular areas of interest. Therefore, a default value of 30 (see Fig. 14) seemed to provide a good balance between these two options in our analyses (this value detects 442 regions for a 3.3% of the total retina, Fig. 14).

4.2. flag_external (line 54 of the code): This parameter controls the overall area to be used as reference for the relative quantification of the vascular and neovascular percentages. When this flag is set to 1 (default), the overall reference area is the calculated area of the whole retina. There are occasions, however, when the user may be interested in quantifying the relative vascular and neovascular areas from a partial image that does not have external retinal borders defined (or the borders are not present within the image). This is the case, for example, in the manufactured images below, where a section of the original image has been removed for further analysis (Fig. 16):

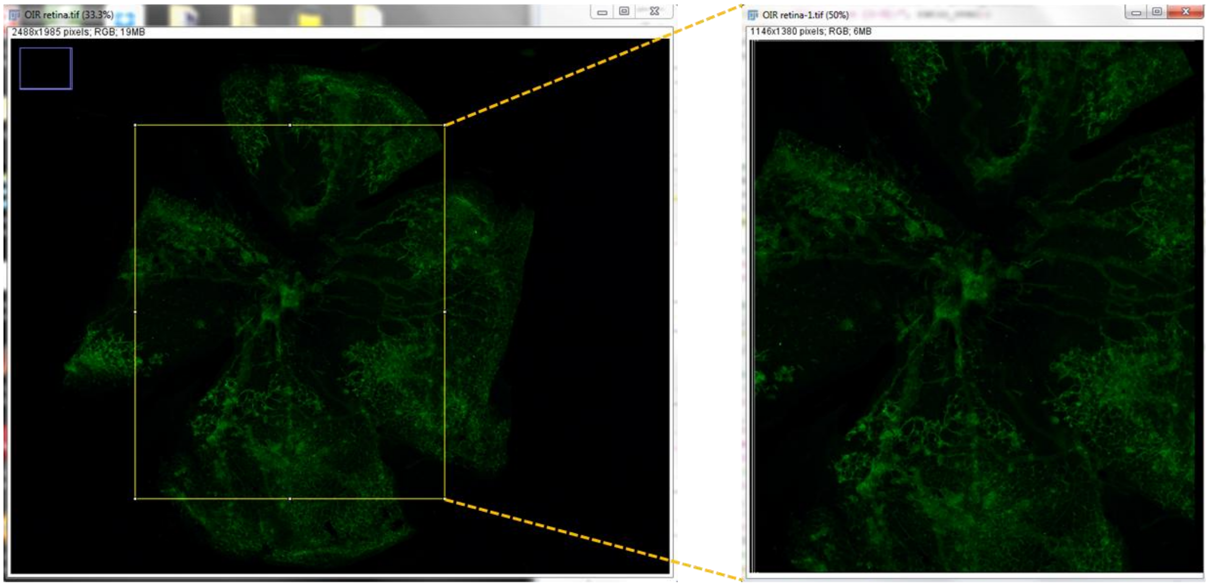

Figure 16. Magnified section (right) taken from the original image (left) to be analysed using a flag_external parameter set to 0

In these cases, it may be more reliable for the program to use the area of the full window as reference for the relative measurements. By setting the parameter flag_external to 0, we tell the software to use the full window as reference for the measurements. In this case, it is also necessary that the first threshold (see section (C) above) is selected so that the white template covers to the full window.

When this is done, the rest of the analysis is as indicated above in the working example: the other 3 thresholds are selected and final images and tables of relative measurements are provided (Fig. 17):

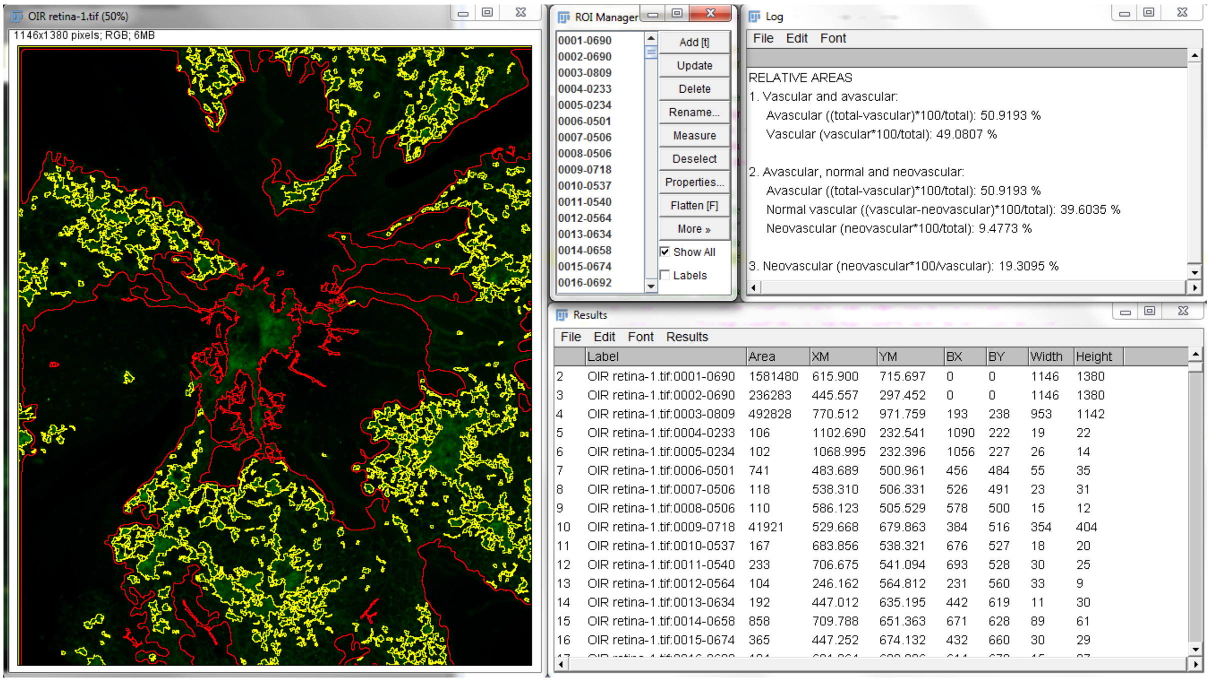

Figure 17. Analysis of partial retina by setting the flag_external parameter to 0

Notice that in this case only red (vascular) and yellow (neovascular) lines are shown.
